# An integrated approach to unravel a crucial structural property required for the function of the insect steroidogenic Halloween protein Noppera-bo

**DOI:** 10.1074/jbc.RA119.011463

**Published:** 2020-04-02

**Authors:** Kotaro Koiwai, Kazue Inaba, Kana Morohashi, Sora Enya, Reina Arai, Hirotatsu Kojima, Takayoshi Okabe, Yuuta Fujikawa, Hideshi Inoue, Ryunosuke Yoshino, Takatsugu Hirokawa, Koichiro Kato, Kaori Fukuzawa, Yuko Shimada-Niwa, Akira Nakamura, Fumiaki Yumoto, Toshiya Senda, Ryusuke Niwa

**Affiliations:** a Structural Biology Research Center, Photon Factory, Institute of Materials Structure Science, High Energy Accelerator Research Organization, 1-1 Oho, Tsukuba, Ibaraki 305-0801, Japan; b Graduate School of Life and Environmental Sciences, University of Tsukuba, 1-1-1 Tennodai, Tsukuba, Ibaraki 305-8572, Japan; c Drug Discovery Initiative, The University of Tokyo, 7-3-1 Hongo, Bunkyo-ku, Tokyo 113-0033, Japan; d School of Life Sciences, Tokyo University of Pharmacy and Life Sciences, 1432-1 Horinouchi, Hachioji, Tokyo 192-0392, Japan; e Graduate School of Comprehensive Human Sciences Majors of Medical Sciences, University of Tsukuba, 1-1-1 Tennodai, Tsukuba, Ibaraki 305-8575, Japan; f Transborder Medical Research Center, University of Tsukuba, 1-1-1 Tennodai, Tsukuba, Ibaraki 305-8575, Japan; g Division of Biomedical Science, Faculty of Medicine, University of Tsukuba, 1-1-1 Tennodai, Tsukuba, Ibaraki 305-8575, Japan; h Molecular Profiling Research Center for Drug Discovery, National Institute of Advanced Industrial Science and Technology, 2-4-7 Aomi, Koto-ku, Tokyo 135-0064, Japan; i Mizuho Information & Research Institute, Inc., 2-3 Kanda Nishiki-cho, Chiyoda-ku, Tokyo 101-8443, Japan; j School of Pharmacy and Pharmaceutical Sciences, Hoshi University, 2-4-41 Ebara, Shinagawa-ku, Tokyo 142-8501, Japan; k Life Science Center for Survival Dynamics, Tsukuba Advanced Research Alliance (TARA), University of Tsukuba, 1-1-1 Tennodai, Tsukuba, Ibaraki 305-8577, Japan; l Institute of Molecular Embryology and Genetics, Kumamoto University, 2-2-1 Honjo, Chuo-ku, Kumamoto 860-0811, Japan; m School of High Energy Accelerator Science, Sokendai University, 1-1 Oho, Tsukuba, Ibaraki 305-0801, Japan; n Faculty of Pure and Applied Sciences, University of Tsukuba, 1-1-1 Tennodai, Ibaraki 305-8571, Japan

**Keywords:** crystal structure, molecular dynamics, Drosophila, steroid hormone, estrogen, lipid metabolism, 17β-estradiol, ecdysone, glutathione S-transferase, GSTE14, insecticide, ecdysteroid

## Abstract

Ecdysteroids are the principal steroid hormones essential for insect development and physiology. In the last 18 years, several enzymes responsible for ecdysteroid biosynthesis encoded by Halloween genes were identified and genetically and biochemically characterized. However, the tertiary structures of these proteins have not yet been characterized. Here, we report the results of an integrated series of *in silico*, *in vitro*, and *in vivo* analyses of the Halloween GST protein Noppera-bo (Nobo). We determined crystal structures of *Drosophila melanogaster* Nobo (DmNobo) complexed with GSH and 17β-estradiol, a DmNobo inhibitor. 17β-Estradiol almost fully occupied the putative ligand-binding pocket and a prominent hydrogen bond formed between 17β-estradiol and Asp-113 of DmNobo. We found that Asp-113 is essential for 17β-estradiol–mediated inhibition of DmNobo enzymatic activity, as 17β-estradiol did not inhibit and physically interacted less with the D113A DmNobo variant. Asp-113 is highly conserved among Nobo proteins, but not among other GSTs, implying that this residue is important for endogenous Nobo function. Indeed, a homozygous *nobo* allele with the D113A substitution exhibited embryonic lethality and an undifferentiated cuticle structure, a phenocopy of complete loss-of-function *nobo* homozygotes. These results suggest that the *nobo* family of GST proteins has acquired a unique amino acid residue that appears to be essential for binding an endogenous sterol substrate to regulate ecdysteroid biosynthesis. To the best of our knowledge, ours is the first study describing the structural characteristics of insect steroidogenic Halloween proteins. Our findings provide insights relevant for applied entomology to develop insecticides that specifically inhibit ecdysteroid biosynthesis.

## Introduction

Ecdysteroids play pivotal roles in regulating many aspects of development and physiology in arthropods, including insects (1, 2). Because ecdysteroids do not exist naturally in animals other than arthropods, it has been long thought that molecules involved in ecdysteroid biosynthesis, secretion, circulation, and reception could be good targets for developing third-generation pesticides that specifically inhibit insect life cycles, with no adverse effects on other animals (3). Thus, the study of ecdysteroids has been important, not only in the basic biological sciences, but also in the field of applied agrobiology.

Ecdysteroids are biosynthesized from dietary sterols that are primarily obtained from food sources (1, 2). The formation of each biosynthetic intermediate going from dietary sterols such as cholesterol to the biologically active form of ecdysteroids, 20-hydroxyecdysone (20E),^5^ is catalyzed by a specific ecdysteroidogenic enzyme (2, 4). Since 2000, a series of these enzymes has been identified. These enzymes include Neverland (5, 6), Non-molting glossy/Shroud (7), Spook/CYP307A1 (8, 9), Spookier/CYP307A2 (9), CYP6T3 (10), Phantom/CYP306A1 (11, 12), Disembodied/CYP302A1 (13), Shadow/CYP315A1 (13), and Shade/CYP314A1 (14). A deficiency of genes encoding these enzymes results in developmental lethality. Particularly in the fruit fly *Drosophila melanogaster,* complete loss-of-function mutants of *shroud*, *spook*, *phantom*, *disembodied*, *shade*, and *shadow*, which are often classified as Halloween mutants, commonly result in embryonic lethality with the loss of differentiated cuticle structures (15). To date, the functions of these enzymes have been characterized genetically, and some of them have also been analyzed biochemically (2, 16). However, none of these enzymes has yet been characterized at the tertiary structure level.

Here, we report the first crystal structure of an ecdysteroidogenic regulator encoded by the Halloween gene, *noppera-bo* (*nobo*) (17–19). *nobo* encodes a member of the epsilon class of cytosolic GSH *S*-transferases (GST, EC 2.5.1.18; hereafter GSTEs) (20). In general, GSTs catalyze various reactions with an activated glutathione (GSH) molecule in the following three ways: GSH conjugation to a substrate, reduction of a substrate using GSH, and isomerization (21). Data from previous studies have demonstrated that *nobo* is specifically expressed in ecdysteroidogenic tissues, including the prothoracic gland and the adult ovary (17–19). Loss-of–*nobo* function mutations in *D. melanogaster* and *Bombyx mori* result in developmental lethality, which are well-rescued by administering 20E (17–19). In addition, the *D. melanogaster* mutants are also rescued by cholesterol, which is the most upstream compound in the ecdysteroid biosynthesis pathway (18). Consistent with the requirement of GSH for GST function, a defect in GSH biosynthesis in *D. melanogaster* also leads to larval lethality, which is partly rescued by the administration of 20E or cholesterol (22). These data indicate that the *nobo* family of GSTs is essential for ecdysteroid biosynthesis by regulating cholesterol trafficking and/or metabolism. However, besides GSH, an endogenous ligand and a catalytic reaction driven by Nobo have not been elucidated.

**Figure 1.**
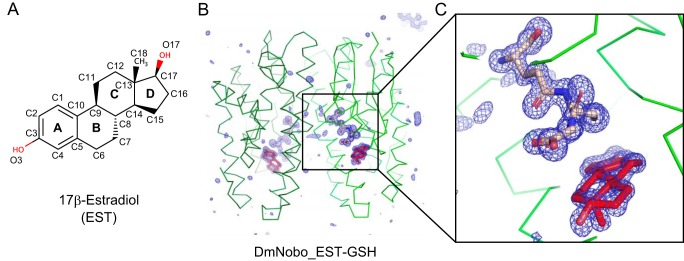
**Crystal structures of the *Drosophila melanogaster* Noppera-bo protein.** *A*, chemical structure of EST. The atoms of the steroid nucleus are indicated. Rings A, B, C, and D are also shown. *B*, simulated annealing omit map for GSH and EST in the DmNobo_EST-GSH complex. An m*F_o_* − D*F_c_* map (*blue*) (4.0σ) is shown. Carbon atoms of DmNobo, GSH, and EST are colored *green*, *wheat*, and *red*, respectively. Oxygen and nitrogen atoms are colored *green* and *blue*, respectively. *C*, an enlarged view of (*B*) around the EST and GSH ligands.

In this study, we utilized the vertebrate female sex hormone 17β-estradiol (EST) (Fig. 1*A*) as a molecular probe to gain insight into Nobo ligand recognition, based on our previous finding that EST inhibits the GSH conjugation activity of *D. melanogaster* Nobo (DmNobo; also known as DmGSTE14) (23). We therefore considered the complex of DmNobo and EST to be an ideal target for elucidating a three-dimensional structure of an ecdysteroidogenic Halloween protein and characterizing the interaction between DmNobo and its potent inhibitor. Moreover, we used an integrated, combined approach based on quantum chemical calculations, molecular dynamics (MD) simulations, biochemical and biophysical analyses, and molecular genetics. Consequently, we identified one DmNobo amino acid residue that is strongly conserved only in the Nobo family of GSTs, which is crucial for DmNobo inhibition by EST and for the normal *in vivo* function of DmNobo during *D. melanogaster* embryogenesis.

## Results

### Crystal structure of DmNobo

The crystal structure of the apo form of DmNobo (DmNobo_Apo) was determined at 1.50-Å resolution by the molecular replacement method (Fig. S1*A* and Table S1). DmNobo forms a polypeptide homodimer with a canonical GST fold, which has a well-conserved GSH-binding site (G-site) and a hydrophobic substrate-binding pocket (H-site) adjacent to the G-site (21, 24). The crystal structures of the DmNobo_GSH, DmNobo_EST, and DmNobo_EST-GSH complexes were also determined at resolutions of 1.75 Å, 1.70 Å, and 1.55 Å, respectively (Fig. 1, *B* and *C*, Fig. S1*B*, and Table S1). The crystal structures of the DmNobo_EST and DmNobo_EST-GSH complexes reproducibly showed clear electron densities for EST. GSH and EST binding did not affect the overall structure of DmNobo (Fig. S1*C*); the root mean square deviation (RMSD) values for each pair among the four crystal structures were comparable with respect to the estimated coordinate errors (Table S2).

GSH, a common substrate of GSTs (21, 24), was found in the G-site of DmNobo. Crystallographic analysis revealed that the position and conformation of GSH in DmNobo and interaction between GSH and DmNobo were essentially identical to those in other GSTEs (25–27). GSH is recognized by an intensive hydrogen bond network with Gln-43, His-55, Val-57, Pro-58, Asp-69, Ser-70, His-71, and Ser-107 in the G-site (Fig. S2). Moreover, these residues are well-conserved among not only GSTEs but also the delta and theta classes of GSTs (hereafter GSTD proteins and GSTT proteins, respectively), which are closely related to GSTEs (Fig. S3, *A* and *B*) (20). Therefore, we conclude that the interaction between the G-site and GSH cannot account for the unique functional property of DmNobo, as compared with other GSTD/E/T proteins.

### Molecular mechanism of EST recognition by DmNobo

EST was bound in the H-site, which has a hydrophobic character. The electron-density map clearly showed that the compound in the H-site was the intact EST molecule (Fig. S1*D*). The EST molecule had no chemical modifications, including reduction and *S*-glutathionylation. The H-site, of which volume is ∼365 Å^3^, was mostly filled with the EST molecule, which has a volume of ∼350 Å^3^, and no space was available to accommodate another compound in the H-site (Fig. S4).

**Figure 2.**
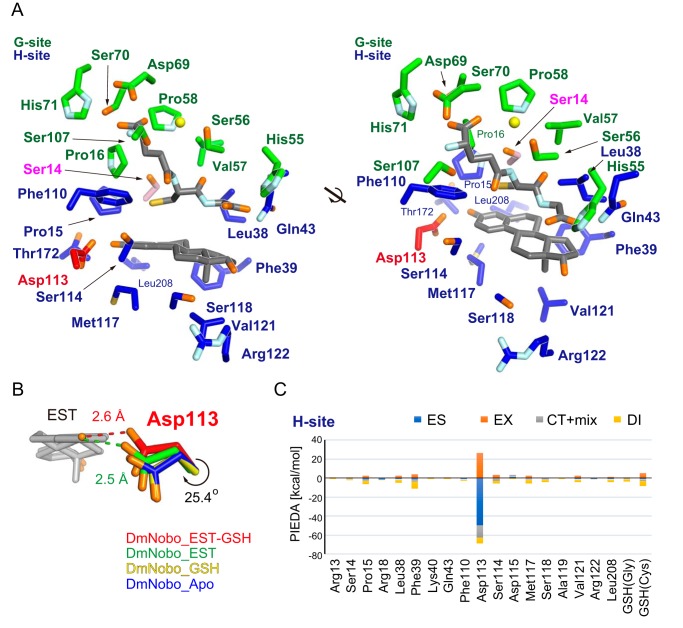
**Asp-113 in the H-site interacts with 17β-estradiol.** *A*, GSH and EST interacting residues. Carbon atoms of the G- and H-sites are colored in *green* and *blue*, respectively. Common residues of the G- and H-sites (Ser-14, Pro-15, Leu-38, Gln-43, and Phe-110) are assigned as those of the H-site in this figure. Carbon atoms in Ser-14, Asp-113, and ligands (GSH and EST) are colored in *pink*, *red*, and *gray*, respectively. A water molecule interacting with each ligand is represented with a *yellow sphere*. The 2 views are related by a 30° rotation around the bold black line axis. *B*, conformational change of Asp-113 upon ligand binding. Carbon atoms in DmNobo_Apo, DmNobo_GSH, DmNobo_EST, and DmNobo_EST-GSH are shown in *blue*, *yellow*, *green*, and *red*, respectively. A hydrogen bond between the O3 atom of EST and Oδ in Asp-113 is indicated by a *dashed line*. The difference in the χ1 torsion angle of Asp-113 between DmNobo_GSH and DmNobo_EST-GSH was 25.4°. *C*, interaction energies between EST and other atoms in the DmNobo_EST-GSH complex. The interaction energies were calculated from the PIEDA analysis, based on the FMO calculation. ES, EX, CT+mix, and DI indicate the electrostatic energy, exchange repulsion energy, charge transfer energy and higher order mixed term, and dispersion energy, respectively. Residues within a distance of twice the van der Waals radii from the EST atoms are shown. Numerical data for (*C*) are available in Table S4.

**Figure 3.**
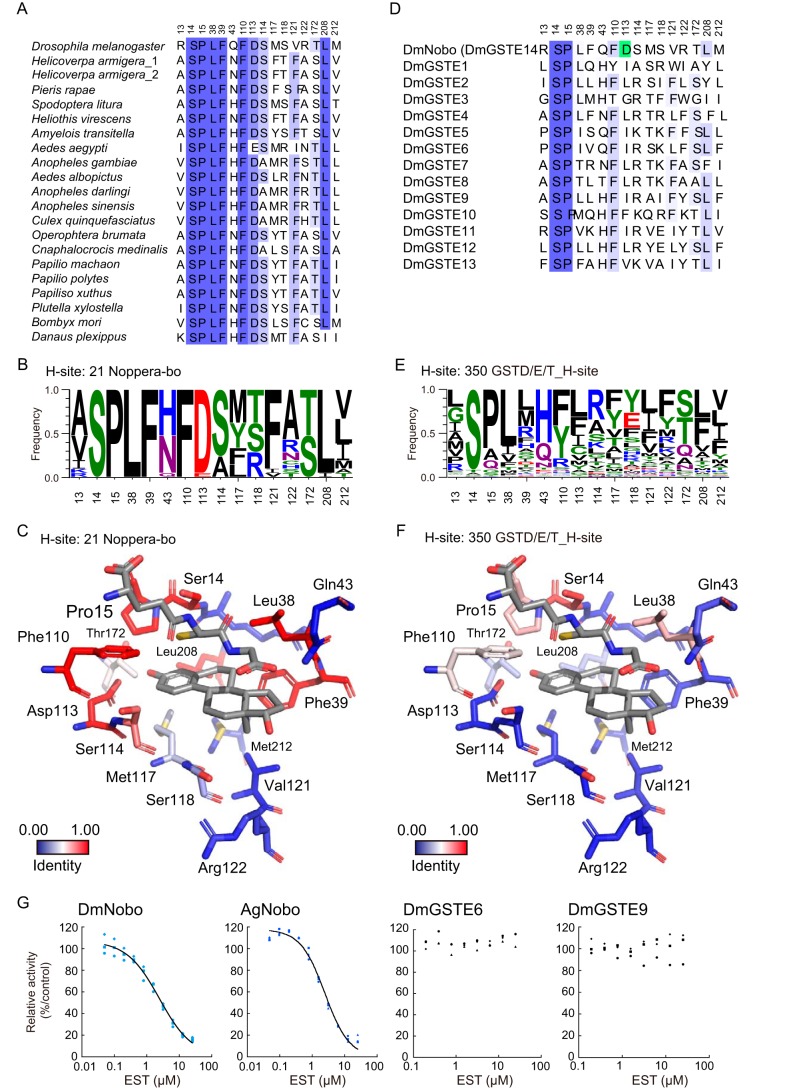
**Consensus amino acid residues in the H-sites of Nobo orthologues.** *A*, amino acid-sequence alignment of the H-site residues of 21 Nobo orthologues. These sequences were aligned using COBALT and manually edited, based on the crystal structure of DmNobo. The accession numbers of *H. armigera*_1 and _2 are XP_021192638.1 and A0A2W1BRE1, respectively. *B*, frequencies of amino acid residues forming the H-sites of 21 Nobo. The frequencies were calculated using LOGO. *C*, conservation ratios of H-site residues among Nobo proteins are mapped to the tertiary structure of DmNobo. *D*, amino acid sequence alignment of the H-site residues of DmGSTE. Asp-113 of DmNobo is colored in *green. E*, frequencies of amino acid residues forming the H-sites of GSTD/E/T proteins. The frequencies were calculated using LOGO. *F*, conservation ratios of H-site residues among GSTD/E/T proteins including Nobo proteins (Fig. S3*A* and Table S2) are mapped to the tertiary structure of DmNobo. *G*, EST-dependent inhibition of the GSH conjugation activities of DmNobo, AgNobo, DmGSTE6, and DmGSTE9. 3,4-DNADCF was used as an artificial fluorescent substrate. Each relative activity is defined as the ratio of activity, when compared with the respective proteins without EST. All of the data points in triplicate assays are indicated. The values of IC_50_ were 2.33 (± 0.08) μm for DmNobo, 2.07 (± 0.36) μm for AgNobo, >25 μm for DmGSTE6, and >25 μm for DmGSTE9.

Of the 16 amino acid residues lining the H-site, Arg-13, Ser-14, Gln-43, Arg-122, and Met-212 do not have direct contacts with EST (Table S3). The D-ring of EST is situated near the entrance of the H-site and exposed to the solvent. Only a few interactions are observed between the D-ring of EST and DmNobo (Fig. 2*A* and Table S3). In contrast, the A-ring of EST is located deep inside of the H-site and makes intensive hydrophobic interactions with H-site residues (Pro-15, Leu-38, Phe-39, Phe-110, Ser-114, Met-117, and Leu-208) (Fig. 2*A* and Table S3). Other amino acid residues interact with other portions of EST, such as Ser-118 at the side of C-ring, Val-121 near C-18, and Thr-172 near O3. These amino acid residues interacting with EST are well-conserved among the Nobo proteins but not among DmGSTD/E/T proteins (Fig. 3, *A–F*, and Table S3). These results suggest that the three-dimensional structure of the H-site, particularly near the A-ring of EST, is conserved in Nobo proteins and has different characteristics from DmGSTD/E/T proteins.

Although the H-site has an overall hydrophobic character, there is one charged residue, Asp-113, in the H-site. Asp-113, which is nearly completely conserved in the Nobo proteins (see below), is located at the innermost region of the H-site. EST binding induces a rotation of the χ1 angle of Asp-113 by 25.4°, and Oδ of Asp-113 forms a hydrogen bond with O3 of EST (Fig. 2*B*). This is the only hydrogen bond found between EST and DmNobo and seems to be critical for EST binding.

To evaluate the contribution of the hydrogen bond to the interaction with EST, total interaction energies between EST fragments and DmNobo amino acid residues were calculated using the fragment molecular orbital (FMO) method, which can evaluate the interfragment interaction energy (IFIE) based on the quantum chemistry (28, 29). The FMO calculation classifies the IFIE into four energy categories, namely the electrostatic energy (ES), exchange-repulsion energy (EX), charge-transfer energy and higher-order mixed term (CT+mix), and dispersion energy (DI). The FMO calculation estimated that the ES represented approximately half of the total IFIE (−41.4 kcal/mol *versus* −82.4 kcal/mol) (Fig. 2*C* and Table S4). The crystal structure suggested that the ES arises from the hydrogen bond between Oδ of Asp-113 and O3 of EST (Table S4). These results suggested that Asp-113 plays a critical role in interacting with EST.

### Asp-113 in DmNobo is essential for EST binding

The importance of the Asp-113–EST hydrogen bond for EST binding was biochemically examined with a recombinant mutated DmNobo protein carrying D113A amino acid substitution (DmNobo D113A). DmNobo D113A lacks the sidechain carboxyl group at position 113 and therefore cannot form a hydrogen bond with EST. The crystal structure of the DmNobo D113A did not show significant structural differences compared with the WT DmNobo (DmNobo WT) protein (Fig. S5, *A* and *B*).

**Figure 4.**
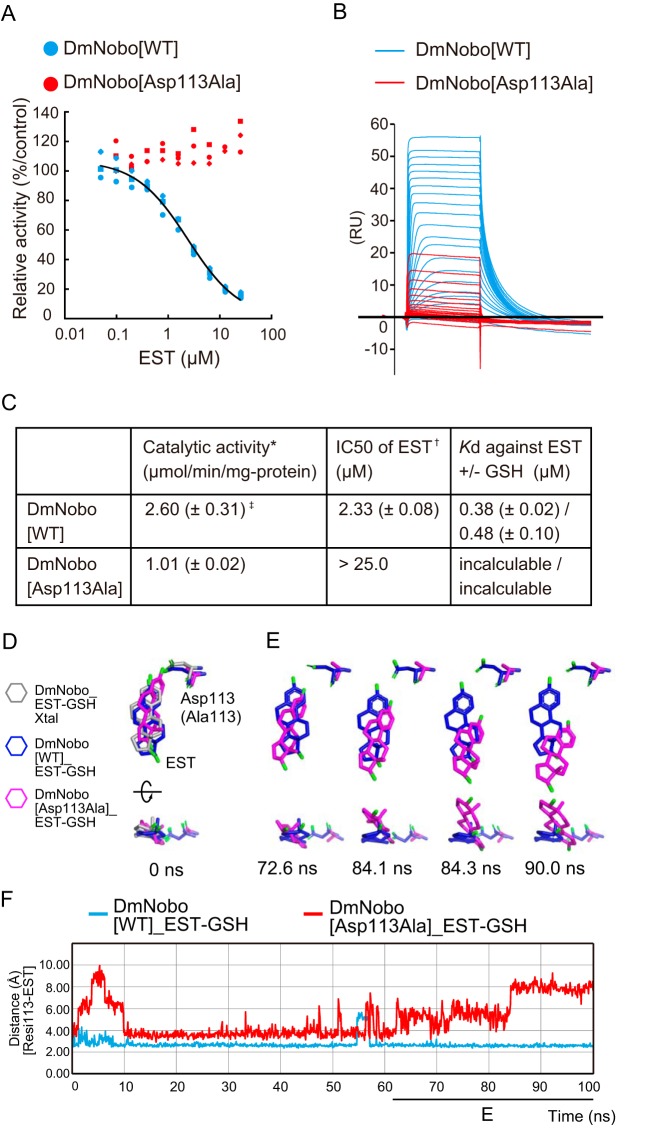
**Asp-113 is essential for DmNobo binding to EST.** *A*, EST-dependent inhibition of the GSH conjugation activity of DmNobo WT (*cyan*) and DmNobo D113A (*red*). 3,4-DNADCF was used as an artificial fluorescent substrate. In each case, the relative activity is defined as the ratio of activity, when compared with DmNobo WT without EST. All of the data points in triplicate assays are shown. *B*, sensorgrams of surface plasmon resonance analysis of DmNobo proteins with EST. DmNobo WT or DmNobo D113A was immobilized to a sensor chip, and solutions containing a series of EST concentrations were applied in presence of 1 mm GSH. *C*, kinetic parameters of DmNobo proteins. Catalytic activity (*) and IC_50_ of EST (†) indicate 3,4-DNADCF-specific GSH conjugation activity and the IC_50_ of EST against 3,4-DNADCF–specific GSH conjugation activity, respectively. Values in parentheses indicate S.D. from triplicate assays (‡). *D–F*, *in silico* evaluation of the contribution of Asp-113 to the interaction between DmNobo and EST. MD simulations of the DmNobo WT or DmNobo D113A complex with EST and GSH in a TIP3P-water model were carried out at 300 K for 100 ns. These simulations were performed in triplicate. *D*, MD models at 0 ns of DmNobo with EST and GSH (*blue*), DmNobo D113A with EST and GSH (*magenta*), and the crystal structure of DmNobo_EST-GSH (EST-GSH_Xtal, *gray*). The *upper models* are shown from above the EST ligand, and the *lower models* are rotated 90° from the upper models. Hydrogen atoms are not shown. *E*, MD models of DmNobo WT_EST-GSH and DmNobo D113A_EST-GSH from 72.6 ns to 90.0 ns. *F*, distance between Oδ of Asp-113 of DmNobo WT or Cβ of DmNobo D113A and the O3 atom of EST at each frame.

We first examined the enzymatic activities of DmNobo WT and DmNobo D113A using an *in vitro* enzymatic assay system with the fluorogenic substrate 3,4-DNADCF (23). In this assay system, GSTs catalyze GSH conjugation to the nonfluorescent molecule, 3,4-DNADCF, giving rise to highly fluorescent product, 4-GS-3-NADCF. In the absence of EST, both DmNobo WT and DmNobo D113A showed GSH conjugation activity (Fig. 4*C*) although the activity of DmNobo D113A decreased by approximately half of DmNobo WT. In the presence of EST, as expected from the EST-binding to the H-site, the enzymatic activity of DmNobo WT was inhibited with an IC_50_ value of ∼2.3 μm (Fig. 4, *A* and *C*). In contrast, the enzymatic activity of DmNobo D113A was not inhibited by EST, even at a concentration of 25 μm (Fig. 4, *A* and *C*).

We next measured the dissociation constant (*K_d_*) values between DmNobo and EST by performing surface plasmon-resonance (SPR) analysis. The *K_d_* values between DmNobo WT and EST in the presence or absence of GSH were 0.38 ± 0.02 μm and 0.48 ± 0.10 μm, respectively (Fig. 4, *B* and *C*). In contrast, it was barely possible to determine the *K_d_* value between DmNobo D113A and EST because of a weak interaction (Fig. 4, *B* and *C*), which was consistent with crystal structure analysis (Fig. S5*C*). These results suggest that Asp-113 is critical for interaction with EST.

We also employed MD simulations to confirm the contribution of Asp-113 to the interaction with EST using DmNobo WT and DmNobo D113A as models. In these MD simulations, the initial structures of EST and the DmNobo proteins were defined based on data acquired from our crystallographic analyses (Fig. 4*D*). Although simulating DmNobo WT for 100 nanoseconds (ns), we found that the distance between Oδ of Asp-113 and the hydroxyl group of EST was relatively constant (Fig. 4, *E* and *F*, and Movies S1 and S2). However, when simulating DmNobo D113A, the distance between Ala-113 and the hydroxyl group of EST increased over time, and EST moved from the initial position (Fig. 4, *E* and *F*, and Movies 1 and 2). Among three independent MD simulations, the maximum RMSD value of EST in DmNobo WT was less than ∼6.60 Å (Fig. S6, *A* and *B*). In contrast, with the MD simulation of DmNobo D113A, the maximum RMSD value was less than ∼9.54 Å (Fig. S6, *A* and *B*). These simulation results also support the possibility that hydrogen bonding between Asp-113 and EST is required for stable binding of EST to the H-site.

### Evolutionary conservation of Asp-113 in Noppera-bo

The *nobo* family of GSTs is well-conserved in Diptera and Lepidoptera (18, 30, 31). Amino acid sequence analysis revealed that all Nobo proteins from 6 dipteran and 13 lepidopteran species have Asp at the position corresponding to Asp-113 of DmNobo (Fig. 3, *A*, *B*, and *D*). An exception is found in Nobo of the yellow fever mosquito *Aedes aegypti*, as the corresponding amino acid residue of *A. aegypti* Nobo is Glu, which also has a carboxyl group in the side chain similar to Asp. In contrast, no Asp/Glu residue was found at the corresponding position of the DmGSTD/E/T proteins, other than Nobo (Fig. 3, *C*, *E*, and *F*). Consistent with the amino acid composition, EST inhibited the enzymatic activity of the African malaria mosquito *Anopheles gambiae* Nobo (AgNobo), but not that of the DmGSTE6 or DmGSTE9 recombinant protein (Fig. 3*G*). Furthermore, as well as DmNobo D113A, a point mutation of AgNobo at Asp-111 to Ala attenuated inhibitory activity of EST against its enzymatic activity (Fig. S7). These results suggest that Nobo proteins utilize Asp-113 to recognize their target compounds as a common feature and that Asp-113 serves a biological role.

### Asp-113 is essential for Drosophila melanogaster embryogenesis

**Figure 5.**
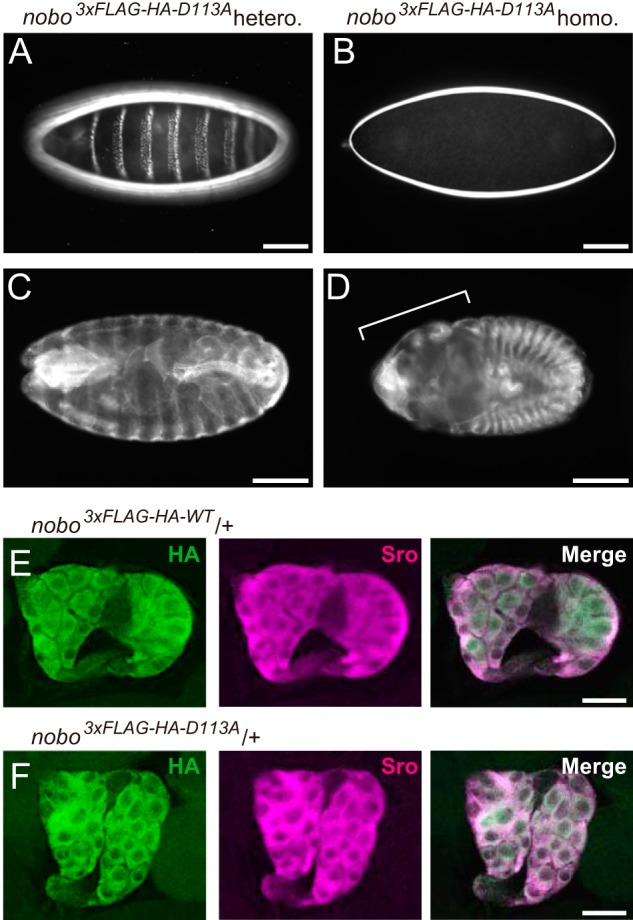
**in vivo analyses of D113A.** *A* and *B*, dark-field images of embryonic cuticles from *nobo^3^*^×^*^FLAG-HA-D113A^* (*A*) heterozygotes (*nobo^3^*^×^*^FLAG-HA-D113A^*/*CyO*) and (*B*) homozygotes (*nobo^3^*^×^*^FLAG-HA-D113A^*/*nobo^3^*^×^*^FLAG-HA-D113A^*). *C* and *D*, anti-FasIII antibody staining to visualize overall embryo morphologies. *C*, *nobo^3^*^×^*^FLAG-HA-D113A^* heterozygotes. *D*, *nobo^3^*^×^*^FLAG-HA-D113A^* homozygotes. The *bracket* indicates defective head involution. *E* and *F*, immunohistochemistry for the ring glands from *nobo^3^*^×^*^FLAG-HA-D113^* (*E*) heterozygous and *nobo^3^*^×^*^FLAG-HA-D113A^* (*F*) heterozygous third-instar larvae. *Green* and *magenta* represent the immunostaining observed with anti-HA and anti-Shroud (*Sro*) antibodies, respectively. Sro was detected as a marker of the prothoracic gland. *Scale bars*, 100 μm for *A–D* and 50 μm for *E* and *F*.

Finally, we examined whether Asp-113 is essential for any *in vivo* biological function of DmNobo. We utilized a CRISPR-Cas9–based knock-in strategy to generate a *nobo* allele encoding a D113A point mutation (*nobo^3^*^×^*^FLAG-HA-D113A^*). We found that no trans-heterozygous mutant *D. melanogaster* with *nobo^3^*^×^*^FLAG-HA-D113A^* and the complete loss-of–*nobo* function allele (*nobo^KO^*) (18) survived to the adult stage (Table 1). By performing a detailed developmental stage analysis, we identified no first-instar larvae or later-staged insects with the *nobo^3^*^×^*^FLAG-HA-D113A^*/*nobo^KO^* genotype. These results indicate that the *nobo^3^*^×^*^FLAG-HA-D113A^*/*nobo^KO^* genotype is embryonic lethal. We also found that *nobo^3^*^×^*^FLAG-HA-D113A^*/*nobo^KO^* embryos exhibit an undifferentiated cuticle phenotype (Fig. 5, *A* and *B*) and a failure of head involution (Fig. 5, *C* and *D*). These phenotypic characteristics were very similar to the feature of Halloween mutants, such as *nobo^KO^*/*nobo^KO^* homozygotes (18). We confirmed that the protein level of Nobo^3×FLAG-HA-D113A^ was comparable to that of Nobo^3×FLAG-HA-WT^ (Fig. 5, *E* and *F*), suggesting that the phenotypes were because of loss of protein function, but not impaired gene expression. Taken together, these results show that Asp-113 of DmNobo serves a biological function in normal development from the embryonic stage to the adult stage.

**Table 1:** **Viability of *nobo*^*3*×*FLAG-HA-D113A*^/*nobo^KO^* knock-in animals** Cy− and Cy+ indicate animals with straight wings and curly wings, respectively. ^†^N.D. indicates not determined.

Background	Knock-in gene	Mating *w*; *nobo^KO^*/CyO-GFP (female) ×	Number of adults Cy− (Cy+)	Number of first instar larvae without GFP (with GFP)
*nobo^KO^*	*nobo^3^*^×*FLAG-HA-WT*^	*w*; *nobo^3^*^×*FLAG-HA-WT*^/CyO-GFP (male)	83 (172)	N.D.^†^
	*nobo^3^*^×*FLAG-HA-D113A*^	*w*; *nobo^3^*^×*FLAG-HA-D113A*^/CyO-GFP (male)	0 (187)	0 (157)

## Discussion

In this study, we employed an integrated experimental approach, involving *in silico, in vitro*, and *in vivo* analyses to unravel the structure-function relationship of the ecdysteroidogenic GST protein, Nobo. GSTs are widely expressed in all eukaryotes and are also massively duplicated and diversified (24). Among them, the Nobo family of GST proteins is strictly required for ecdysteroid biosynthesis in insects. Importantly, the lethality of *nobo* mutation in *D. melanogaster* is rescued by overexpressing *nobo* orthologues, but not by overexpressing non–*nobo*-type *gst* genes involved in detoxification and pigment synthesis (18). This fact strongly indicates that, when compared with canonical GSTs, Nobo proteins must possess a unique structural property that makes Nobo specialized for ecdysteroid biosynthesis. Regarding this point, this study is significant in that we found that the unique acidic amino acid, Asp/Glu-113, is crucial for the *in vivo* function of Nobo. It should be noted that, besides Asp/Glu-113, other amino acids constituting the H-sites are also highly conserved among 21 Nobo proteins (Fig. 3, *A*, *B*, and *D*). These common features imply that the Nobo proteins might share an identical endogenous ligand for the H-site in the ecdysteroidogenic tissues among the species.

An endogenous ligand for Nobo remains a mystery. This study, however, provides some clues for considering candidates for an endogenous ligand. First, it is very likely that the ligand forms a hydrogen bond with the Oδ/Oϵ atom of Asp/Glu-113, given that the *nobo* D113A point mutation was embryonic lethal and the complete loss-of-function *nobo* phenocopy in mutant *D. melangaster*. Second, considering the complementary shape between the H-site and EST, it seems reasonable to predict that the endogenous ligand(s) is at least similar in shape to steroids. This prediction is also supported by the fact that Nobo acts in ecdysteroidogenic tissues where steroidal molecules are enriched. One steroid that possesses these features is cholesterol. Evidence from our previous study suggests that *nobo* may be involved in cholesterol transport and/or metabolism in ecdysteroidogenic tissues (17–19). Very interestingly, an MD simulation indeed predicted that cholesterol can stably bind to the H-site of DmNobo via a hydrogen bond between the hydroxyl group of cholesterol (C3 position) and Asp-113 of DmNobo (Fig. S8). However, paradoxically, it seems that cholesterol contains no site for a chemical reaction with GSH by DmNobo. It is possible that Nobo might serve as a carrier or a transporter for the ligand in cells, possibly cholesterol, as several classes of GSTs have been shown to exhibit “ligandin” function (32), which might be an initial step of the ecdysteroid biosynthesis pathway. Currently, we have failed in multiple attempts to detect DmNobo-cholesterol complexes via crystallographic analyses, and further experiments are needed for clarify any interaction between Nobo and cholesterol.

The activities of insect ecdysteroids can be disrupted *in vivo* using chemical agonists and antagonists of the ecdysone receptor, some of which are also utilized as insecticides (33). However, chemical compounds that specifically inhibit ecdysteroid biosynthesis are not available. This study provides the first structural information for guiding the development of efficient Nobo inhibitors, which might serve as seed compounds for new insecticides in the future. However, it should be noted that EST and estrogenic chemical compounds are often recognized as dangerous endocrine-disrupting chemicals against wild animals (34). Therefore, although EST is a prominent inhibitor of Nobo, a practical compound that can be utilized as an actual insecticide must display no estrogenic activity. To consider this problem, it is important to note a difference in the EST-recognition patterns between DmNobo and the mammalian estrogen receptor alpha (ERα) protein (35–38). The details of the EST-ERα interaction were investigated using the crystal structures of human ERα in an EST-bound form (35, 39). In ERα, Glu-353 interacts with the O3 atom of EST, Phe-404 interacts with the A-ring of EST via a CH/π interaction, His-524 interacts with the O17 atom of EST, and hydrophobic residues interact with the steroid nucleus. Each of these recognition patterns were found in DmNobo such as a hydrogen bond between Asp-113 and O3 atom of EST and an SH/π interaction between Cys residue of GSH and the A-ring of EST, except for a hydrogen bond with the O17 atom of EST (Fig. S9). Given this difference, we expect that a Nobo-specific, nonestrogenic chemical compound can be developed. Currently, we are pursuing large-scale computational calculations to select chemical compounds that satisfy those conditions and an *in vitro* enzymatic assay to examine DmNobo inhibition.

We emphasize that this report is the first to describe the physical interactions between a Halloween protein and a potent inhibitor at the atomic level. Our interdisciplinary approach will also be applicable for Nobo proteins other than *D. melanogaster*, such as disease vector mosquitos and the agricultural pest moths, and might be a viable strategy for developing new insecticides useful for human societies.

## Experimental procedures

### Protein expression and purification

The protein-expression plasmid pCold-III (Takara Bio Inc., Kusatsu, Japan) was used to express recombinant GST proteins in *Escherichia coli*. Coding sequences (CDSs) of *D. melanogaster nobo* (*CG4688, Dmnobo*), *gste6* (*CG17530, Dmgste6*), *gste9* (*CG17534, Dmgste9*), and *A. gambiae gste8* (*AGAP009190*, *Agnobo*) were amplified by the polymerase chain reaction (PCR) using complementary DNA derived from *D. melanogaster* larvae and *A. gambiae* larvae. The primers used for PCR were nobo, forward (5′-CAGTCATATGATGTCTCAGCCCAAGCCGATTTTG-3′), nobo, reverse (5′-CTCGAGCTACTCCACCTTCTCGGTGACTACCG-3′), GSTe6, forward (5′-CATATGATGGTGAAATTGACTTTATACGG-3′), GSTe6, reverse (5′-TCTAGATCATGCTTCGAATGTGAAATT-3′), GSTe9, forward (5′-CATATGATGGGAAAATTAGTACTGTACGG-3′), GSTe9, reverse (5′-TCTAGATTACACAATCTTTGTGATCTTCG-3′), agnobo, forward (5′-GGTACCATGATTCTGTACTACGACGAGGTCAGC-3′), and agnobo, reverse (5′-AAGCTTCTACAGCTTAATCTTTCCCGCTAAATG-3′). The *nobo* CDS was subcloned between the NdeI and XhoI restriction enzyme sites in pCold-III to generate the pCold-III_DmNobo WT vector. The *gste6* and *gste9* CDSs were subcloned between the NdeI and XbaI sites in pCold-III. It should be noted that pCold-III added a translation enhancing element (MNHKV) at the N terminus of each of DmNobo, AgNobo, DmGSTE6, and DmGSTE9 proteins.

Expression vectors for DmNobo D113A and AgNobo D111A were constructed by inverse PCR–based site-directed mutagenesis. The entire pCold-III_DmNobo WT and pCold-III_AgNobo WT plasmids were amplified by inverse PCR using a KOD-Plus-Mutagenesis Kit (Toyobo Co., Ltd, Osaka, Japan) using pairs of the oligonucleotides, 5′-CCAGTGATTTTATGTCGGCGATTGTCCGCC-3′ and 5′-CACGTCGGAACAAAAAGGAGCATTCGAAGA-3′ for DmNobo D113A, and 5′-CGCTGCGGAAGTTATGCGTAAAATC-3′ and 5′-CGCTGAAACAAACAGCCGTTGTTG-3′ for AgNobo D111A, as amplification primers. The *E. coli* strain DH5α was transformed with the DpnI-digested PCR products. The plasmids were purified using a FastGene Plasmid Mini Kit (NIPPON Genetics Co., Ltd., Tokyo, Japan). Those DNA sequences were confirmed by Sanger sequencing with one of the following sequencing primers: 5′-ACGCCATATCGCCGAAAGG-3′ or 5′-GGCAGGGATCTTAGATTCTG-3′.

DmNobo, AgNobo, *D. melanogaster* GSTE6 (DmGSTE6), and *D. melanogaster* GSTE9 (DmGSTE9) were expressed in the *E. coli* strain BL21(DE3) (Merck) and purified via GSH-affinity column chromatography, followed by size-exclusion column chromatography. *E. coli* BL21(DE3) cells were transformed with the plasmids, and then the transformed cells were cultured in LB medium supplemented with 50 μg/ml ampicillin at 37 °C. When the *A*_600_ of the culture reached ∼0.6, protein expression was induced with 0.3 mm isopropyl β-d-1-thiogalactopyranoside. The *E. coli* cells were cultured at 18 °C overnight and then harvested. The harvested cells were suspended in lysis buffer (300 mm NaCl, 25 mm Tris-HCl, pH 8.0, 1 mm CHAPS, 1 mm DTT) and lysed for 2 min by sonication using a VP-305 Ultra 5 Homogenizer (TAITEC), using an output of 7 and a duty of 40%. The lysate was fractionated by centrifugation at 15,000 × *g* for 30 min at 4 °C, and the supernatant was applied to a GSH-affinity column containing a 10-ml bed volume of GSH Sepharose 4B (GE Healthcare). After the column was washed with lysis buffer, the proteins were eluted with 50 ml of elution buffer (140 mm NaCl, 25 mm Tris-HCl, pH 8.0, 1 mm CHAPS, 1 mm DTT, 10 mm GSH). The eluent for DmNobo D113A was concentrated to 2 ml and fractionated with a Superdex 200 increase 10/300 size-exclusion column (GE Healthcare) connected to an ÄKTA FPLC system (GE Healthcare) or those for DmNobo WT, DmGSTE6, DmGSTE9, AgNobo WT, and AgNobo D111A were concentrated to 5 ml and fractionated with HiLoad Superdex 200 16/600 column (GE Healthcare) (Fig. S10, *A* and *B*). The columns were equilibrated with a buffer (150 mm NaCl, 25 mm Tris-HCl, pH 8.0, 1 mm DTT). DmNobo D113A protein was eluded with the same buffer at a flow rate of 0.2 ml/min and others were eluted at a flow rate of 1.0 ml/min. The purity and quality of final products were validated by SDS-PAGE and Coomassie Brilliant Blue staining (Fig. S10*C*). The peak fractions were concentrated to 15 mg/ml and stored at −80 °C. The protein concentrations of DmNobo, DmGSTE6, DmGSTE9, and AgNobo were measured with a NanoDrop ND-1000 spectrophotometer (Thermo Fisher Scientific) using extinction coefficients (ϵ_280_) of 0.671·M^−1^·cm^−1^, 1.274·M^−1^·cm^−1^, 1.128·M^−1^·cm^−1^, and 1.100·M^−1^·cm^−1^, respectively.

### Crystallization

The Protein Crystallization System (40) was used for the initial crystallization screening of DmNobo (16). In total, 384 conditions were examined using the Crystal Screen 1 & 2, Index, PEG/Ion, or PEG/Ion 2 kits from Hampton Research (Aliso Viejo, CA), or the Wizard I & II kit from Molecular Dimensions (Suffolk, UK). DmNobo was crystallized at 20 °C in the presence of 25% (w/v) PEG 3350 in 100 mm Bis-Tris (pH 5.5; index 42), or 45% (v/v) PPG 400 in 100 mm Bis-Tris (pH 6.5; index 58). The crystallization conditions were optimized by changing the pH and the concentration of the precipitation agent, resulting in two types of crystals, DmNobo I and II. DmNobo I crystals were obtained from a buffered solution containing 27.5% (w/v) PEG 3350 in 100 mm MES-NaOH (pH 5.4), and DmNobo II crystals were obtained from a buffered solution containing 42.5% (v/v) PPG400 in 100 mm Bis-Tris (pH 6.4). Crystals of substrate complexes were prepared by soaking the DmNobo II crystals in an artificial mother liquor (42.5% (w/v) PPG 400 in 100 mm Bis-Tris, pH 6.4) containing 10 mm EST, with or without 1 mm GSH, for 6 h.

### Crystal structure determinations

Crystals were picked up with proper size of MicroLoops (MiTeGen, New York), flash frozen in liquid nitrogen, and packed in Uni-Pucks (Molecular Dimensions). Diffraction data were collected at beamline BL-1A in the Photon Factory (Tsukuba, Japan) and at beamline X06SA in the Swiss Light Source. The diffraction datasets collected at the Photon Factory were automatically processed and scaled using XDS (41), POINTLESS (42), and AIMLESS (43) on PReMo (44), and those collected at the Swiss Light Source were processed and scaled using XDS and AIMLESS. Crystallographic statistics are summarized in Table S1.

Phases for DmNobo_Apo_1 (PDB ID: 6KEL) data collected from DmNobo I crystals were determined by the molecular replacement method with MOLREP (45) using the crystal structure of DmGSTE7 (PDB ID = 4PNG) as a search model. Other crystal structures were determined by the molecular replacement method using the crystal structure of DmNobo_Apo_1 as a search model. Molecular models were initially refined with REFMAC5 (46). The models were manually built using COOT (47) and further refined with PHENIX.REFINE (48) repeatedly. The C-terminal four residues could not be modeled because of poor electron density. In this study, the crystal structure of DmNobo_Apo_2 (PDB ID: 6KEM) determined with a DmNobo II crystal was used as the DmNobo_Apo structure when making comparisons with other crystal structures. m*F_o_* − D*F_c_* omit maps for ligands were calculated using PHENIX.REFINE with a simulated annealing protocol. Interactions between DmNobo and GSH or EST were analyzed using PISA (49). The volume of the cavity in DmNobo was calculated using the Channel Finder program in 3V (50), with 4-Å radius for the outer probe and a 1-Å radius for the inner probe. The volumes of GSH or EST were calculated using the Volume Assessor program in 3V, with a 2-Å radius for each probe. The RMSD from a least squares fitting among the DmNobo structures was calculated with GESAMT (51). Atom pairs within a 4.0-Å distance were defined as making direct contacts. All molecular graphics were prepared using the PyMOL Molecular Graphics System, version 1.7.6 (Schrödinger, NY).

### In vitro GST assay

*In vitro* GST assays with 3,4-DNADCF were performed as described previously (23). The stock solutions of DmNobo WT and DmNobo D113A were 200 ng/ml each in solution A (2 mm GSH, 100 mm sodium phosphate buffer, pH 6.5, 0.01% Tween 20). Decreasing concentrations of DmNobo WT and DmNobo D113A, ranging from 200 ng/ml to 0.19 ng/ml, were prepared by 2-fold serial dilution with solution A. The DmNobo dilution series was mixed with an equal volume of solution B (100 mm sodium phosphate buffer, pH 6.5, with 2 μm 3,4-DNADCF in 0.2% DMSO as a co-solvent) in each well of a 96-well plate to initiate the catalytic reaction of DmNobo. The GSH-conjugated product was excited at 485 nm, and the fluorescence intensity at 535 nm (*F*_measured_) was measured every 30 s for 20 min with an infinite 200 PRO instrument (Tecan, Zurich, Switzerland). The fluorescence intensity (*F*_t_) in the reaction mixture without DmNobo (*F*_bg_) was subtracted as the background signal (*F*_t_ = *F*_measured_ − *F*_bg_). The maximum fluorescence intensity (*F*_max_) was the fluorescence intensity that was reached as a plateau. The amount of product in each well (*P*_t_) at the measured time (t) was calculated as *P*_t_ (μmol) = *F*_t_/*F*_max_ × 200 μl × 1 μmol/liter. The rate of product formation (*P*_rate_, μmol/min) was obtained by linear least squares fitting between *P*_t_ and t. The specific activity of DmNobo (μmol/min/mg protein) was defined as *P*_rate_/[protein concentration]. The assay was performed in triplicate.

### GST activity-inhibition assay

EST was dissolved in DMSO to a concentration of 2.5 mm. The 2.5 mm EST solution was diluted to 50 μm EST in solution C (2 mm GSH, 100 mm sodium phosphate buffer, pH 6.5, 0.01% Tween 20, 2% DMSO, and 50 ng/ml DmNobo WT, 50 ng/ml DmNobo D113A, 100 ng/ml AgNobo WT, 100 ng/ml AgNobo D111A, 35 ng/ml DmGSTE6, or 300 ng/ml DmGSTE9). A dilution series of EST, ranging from 50 to 0.19 μm, was prepared by 2-fold serial dilution with solution C. One hundred microliters of each EST solution in the dilution series was mixed with an equivalent amount of solution B in each well of a 96-well plate. *F*_measured_ values were measured after 3 min, as described under “*In vitro* GST assay.” The fluorescence intensity detected in the absence of EST and DmNobo (*F*_bg_) was subtracted as the background in all experiments (*F* = *F*_measured_ − *F*_bg_). *F* at 0 s (*F_0_*) was subtracted from *F* at the measured time (s) (*F*_t_ = *F* − *F*_0_).

The relative activity was calculated as *F*_30_[I]_/*F*_30_[0]_, where [I] and [0] indicate the EST concentrations. The relative activity was plotted against each EST concentration. A fitting curve was calculated based on a plot generated from the following equation when IC_50_ and Hill constant (*n*) were approximated as 1.00 and 1.00, respectively, using KaleidaGraph version 4.5.1 (Synergy Software, Reading, PA): Relative activity (%) = 1/(1 + ([EST]/IC_50_)*^n^*)) × 100. The IC_50_ value was estimated based on the fitting curve. The assay was performed in triplicate.

### Phylogenetic analysis

Nineteen amino acid sequences of DmNobo or *B. mori* Nobo orthologues were found using BLASTP (52) to search the NCBI nonredundant protein database. In addition, a Nobo orthologue in *Helicoverpa armigera* was found in the UniProt Knowledgebase. The accession numbers were XP_021192638.1 for *H. armigera* GSTE14-like isoform X2, A0A2W1BRE1 for *H. armigera* uncharacterized protein, XP_022126447.1 for *Pieris rapae* GSTE14-like, XP_022837694.1 for *Spodoptera litura* GSTE14-like isoform X2, PCG75296.1 for *Heliothis virescens* hypothetical protein B5V51_11931, XP_013196516.1 for *Amyelois transitella* GST1, XP_001658748.2 for *A. aegypti* GSTE14, XP_319963.1 for *A. gambiae* GSTE8, KXJ68754.1 for *Aedes albopictus* hypothetical protein RP20_CCG001852, ETN60212.1 for *Anopheles darlingi* GSTE, KFB39334.1 for *Anopheles sinensis* AGAP009190-PA-like protein, XP_001868776.1 for *Culex quinquefasciatus*, KOB78695.1 for *Operophtera brumata* GST, AIL29314.1 for *Cnaphalocrocis medinalis* GSTE5 partial region, XP_014368559.1 for *Papilio machaon* GSTE14-like, XP_013137131.1 for *Papilio polytes* GST1–1-like, NP_001299034.1 for *Papilio xuthus* GST1–1, NP_001292431.1 for an uncharacterized protein *Plutella xylostella*, ABY66602.1 for *B. mori* GSTE14, and OWR47941.1 for *Danaus plexippus*. Two *nobo* orthologues were found for *H. armigera* in the database.

For phylogenetic analysis of insect GSTD/E/T proteins, previously described amino acid sequences were obtained from the UniProt Knowledgebase, NCBI protein database, and MonarchBase (18, 53–55). Amino acid sequences (503) were aligned with COBALT (56), and the resulting sequence alignment was used for cluster analysis with CLANS (57). A major cluster included 372 amino acid sequences, including those of GSTD/E/T proteins and other GST proteins (Table S5). A phylogenetic tree was drawn with COBALT, using the 372 GSTs and a neighbor-joining algorithm. We identified 371 sequences with a Grishin-sequence difference of 0.9, including 151 GSTDs, 178 GSTEs, and 42 GSTTs. We also identified 21 Nobo proteins among the GSTEs.

To calculate the amino acid frequencies, the obtained alignment was manually edited based on the known crystal structures, using Jalview (58). The amino acid frequencies were calculated and illustrated with WebLOGO version 3.7.4 (59).

### SPR assay

Surface plasmon resonance was measured at 25 °C using Biacore T200 instrument with a CM5 sensor chip (GE Healthcare). DmNobo WT or the DmNobo D113A protein was used as a ligand, and EST was used as an analyte in PBS containing 1% DMSO, in the presence or absence of 1 mm GSH as a running buffer.

The Biacore T200 system with a CM5 sensor chip was filled with the running buffer. The ligands were immobilized on the activated CM5 sensor chip in an acetate buffer (pH 5.0) using a purchased amine-coupling kit (GE Healthcare) to reach 6500 resonance units. The same process was performed in the absence of proteins in one lane on the chip as a background lane.

An EST dilution series was prepared by serial dilution. An EST stock solution (100 mm EST in DMSO) was diluted with running buffer to a concentration of 20 μm. The 20 μm EST solution was serially diluted by two thirds with running buffer 17 times, and the running buffer in the absence of EST was used as the 0-μm EST sample. The analyte was flowed onto the sensor chip for 60 s and allowed to dissociate for 180 s.

EST concentrations of 20.000, 13.333, 8.889, 5.926, 3.951, 2.634, 1.756, 1.171, 0.780, 0.520, 0.347, 0.231, 0.154, 0.103, 0.069, 0.046, 0.030, 0.020, and 0.014 μm were used to calculate its *K_d_*. The background was subtracted from the sensorgrams of the protein-immobilized lanes (sensorgrams shown in Fig. 3*B*). The *K_d_* values of EST for DmNobo WT and DmNobo D113A were evaluated with Biacore T200 Evaluation Software, using data from triplicate assays.

### FMO calculations

*Ab initio* FMO calculations (60–62) were performed on the crystal structures of the DmNobo_Apo, DmNobo_EST-GSH, DmNobo_GSH, and DmNobo_EST complexes. Although DmNobo is a homodimer, only the monomeric structure was utilized for the FMO calculations. Intersubunit interactions were therefore neglected in this study. The crystal structures were modified before performing the FMO calculations. First, all crystal water molecules, except for one that interacts with the carbonyl oxygens of Glu in GSH and Pro-58, and the Oγ atom of Ser-56 of DmNobo (Fig. 2*A*, water in *yellow*), were deleted from the crystal structures. Second, assignment of the protonation state and the addition of hydrogen atoms were performed using the Protonate 3D function of the Molecular Operating Environment program package (Chemical Computing Group, Montreal, Canada). Note that the carboxyl group of Asp-113 was assigned to be ionized. Then, energy minimization of hydrogen atoms was performed with the Amber10:EHT force field. The protonated states of His-55 and His-71 were assumed to be positively charged to form hydrogen bonds with GSH. Then, FMO calculations for the monomeric DmNobo structures were performed using ABINIT-MP software (63, 64). The second-order Møller-Plesset perturbation (MP2) (65, 66) method was used with the 6–31G* basis function as a theoretical calculation level; namely, the FMO-MP2/6–31G* level of theory was used. For the FMO calculations, DmNobo proteins and GSH were fragmented into amino acid units at bonds between the C and Cα atoms of the main chain. Each EST and water molecule was treated as a single fragment. The fragmentation treatment makes it possible to easily calculate the electronic structure of the whole complex and the IFIEs. The obtained IFIEs were further decomposed into four energy components, *i.e.* the ES, EX, CT+mix, and DI components, using PIEDA (28, 29).

### MD simulations

The structures of DmNobo WT_EST-GSH, DmNobo D113A_EST-GSH, and DmNobo_cholesterol-GSH were processed to assign bond orders and hydrogenation. The ionization states of EST, cholesterol, and GSH at pH 7.0 ± 2.0 were predicted using Epik (67), and H-bond optimization was conducted using PROPKA (68). Energy minimization was performed in Maestro using the OPLS3 force field (69).

Preparation for MD simulations was conducted using the Molecular Dynamics System Setup Module of Maestro (Schrödinger, NY). DmNobo WT_EST-GSH and DmNobo D113A_EST-GSH were subjected to energy minimization and placed in an orthorhombic box with a buffer distance of 10 Å to create a hydration model, and the TIP3P water model (70) was used for the hydration model. NaCl (0.15 m) served as the counter ion to neutralize the system.

The MD simulations were performed using Desmond software, version 2.3 (Schrödinger, NY). The cutoff radii for van der Waals and the time step, initial temperature, and pressure of the system were set to 9 Å, 2.0 femtoseconds, 300 K, and 1.01325 bar, respectively. The sampling interval during the simulation was set to 10 picoseconds. Finally, we performed MD simulations using the NPT ensemble for 100 ns.

### Transgenic D. melanogaster insects and genetics

*D. melanogaster* flies were reared on standard agar-cornmeal medium at 25 °C under a 12 h/12 h light/dark cycle. The strain harboring the D113A point mutation (*nobo^3^*^×^*^FLAG-HA-D113A^*), as well as the control WT strain (*nobo^3^*^×^*^FLAG-HA-WT^*), was generated using a CRISPR-Cas9-mediated knock-in strategy (71). Briefly, in each case, the genome of the starter *yw* strain was cut at two sites around the *nobo* locus, and then homologous recombination occurred with appropriate plasmids carrying 5′- and 3′-homology arms and an N-terminal 3× FLAG-HA epitope tag. The pDCC6 plasmid was used for simultaneous expression of both the Cas9 gene and guide RNA (72). The following primer pairs were annealed and then ligated to *Bbs* I-digested pDCC6, which led to the production of three different guide RNA plasmids: 5′-CTTCGTTGGGCTGAGACATTAAGTT-3′ and 5′-AAACAACTTAATGTCTCAGCCCAAC-3′ for Cutter#1, 5′-CTTCGTTACGACGAGCGCAGTCCGC-3′ and 5′-AAACGCGGACTGCGCTCGTCGTAAC-3′ for Cutter#2, and 5′-CTTCGCCGACGTGACAGTGATTTTA-3′ and 5′-AAACTAAAATCACTGTCACGTCGGC-3′ for Cutter#3. The pUC19-based plasmids carrying the homology arms and epitope tags, designated pDonor[KI]-{CG4688_LA}:{3×FLAG/HA/nobo}:{CG4688_RA} and pDonor[KI]-{CG4688_LA}:{3×FLAG/HA/nobo*D113A}:{CG4688_RA}, respectively, were artificially synthesized by VectorBuilder, Inc (Chicago). The entire DNA sequence of each plasmid is shown in Fig. S11 and Fig. S12. To generate the *nobo^3^*^×^*^FLAG-HA-WT^* strain, the Cutter#1, Cutter#2, and pDonor[KI]-{CG4688_LA}:{3×FLAG/HA/nobo}:{CG4688_RA} plasmids were injected into *yw* embryos. To generate the *nobo^3^*^×^*^FLAG-HA-D113A^* strain, the Cutter#1, Cutter#3, and pDonor[KI]-{CG4688_LA}:{3×FLAG/HA/nobo*D113A}:{CG4688_RA} plasmids were injected to *yw* embryos. The proper knock-in strains were identified and characterized, essentially as described previously (73). DNA sequences surrounding the knock-in regions were confirmed by Sanger sequencing.

We found that *nobo^3^*^×^*^FLAG-HA-WT^* homozygous flies were fully viable, whereas *nobo^3^*^×^*^FLAG-HA-D113A^* homozygous flies displayed embryonic lethality. We utilized *nobo^3^*^×^*^FLAG-HA-D113A^* heterozygous and homozygous embryos for cuticle preparation and immunostaining. To formally rule out the possibility that the embryonic lethality was because of anonymous deleterious mutations other than *nobo^3^*^×^*^FLAG-HA-D113A^*, we counted the number of trans-heterozygous flies with a *nobo* knockout (*nobo^KO^*) from a previous report (18), as follows. Heterozygous *nobo^3^*^×^*^FLAG-HA-WT^*, *nobo^3^*^×^*^FLAG-HA-D113A^*, and *nobo-*knockout (*nobo^KO^*) alleles were balanced with *CyO* carrying *Actin5C:gfp* cassette (*CyO-GFP*). Either *nobo^3^*^×^*^FLAG-HA-WT^/CyO-GFP* flies or *nobo^3^*^×^*^FLAG-HA-D113A^/CyO-GFP* flies were crossed with *nobo^KO^/CyO-GFP* flies. The trans-heterozygous flies (*nobo^3^*^×^*^FLAG-HA-WT^/nobo^KO^* or *nobo^3^*^×^*^FLAG-HA-D113A^/nobo^KO^*) should exhibit no GFP signals. We found that GFP-negative *nobo^3^*^×^*^FLAG-HA-WT^/nobo^KO^* embryos hatched normally and developed into adults without any abnormalities, whereas *nobo^3^*^×^*^FLAG-HA-D113A^/nobo^KO^* embryos did not.

### Cuticle preparation and immunostaining

Embryonic cuticle preparation was performed as described previously (74). Immunostaining for whole-mount embryos was conducted as described previously (18). A mouse anti-FasIII mAb 7G10 (1:20 dilution) (Developmental Studies Hybridoma Bank, Iowa City, IA) and an anti-mouse IgG antibody conjugated with Alexa Fluor 488 (1:200 dilution) (Life Technologies) were used for immunostaining the embryos. For immunostaining of the brain-ring gland complex in third-instar larvae, we first crossed *nobo^3^*^×^*^FLAG-HA-WT^* homozygous females or *nobo^3^*^×^*^FLAG-HA-D113A^*/*CyO-GFP* females with Oregon-R WT males. Third-instar larvae of the heterozygous offspring (*nobo^3^*^×^*^FLAG-HA-WT^/*+ or *nobo^3^*^×^*^FLAG-HA-D113A^/*+) were dissected and then immunostained as described previously (75). The antibodies used for the brain-ring gland complex included a rat anti-HA high-affinity mAb (3F10, 1:20 dilution) (Roche), a guinea pig anti-Shroud antibody (76) (1:200 dilution), an anti-rat IgG antibody conjugated with Alexa Fluor 488 (1:200 dilution) (Life Technologies), and an anti-guinea pig IgG antibody conjugated with Alexa Fluor 555 (1:200 dilution) (Life Technologies). Fluorescence images were obtained using an LSM700 microscope (Carl Zeiss).

### Data availability

The X-ray data and coordinates presented in this paper were deposited in the Protein Data Bank under the following PDB IDs: 6KEL, 6KEM, 6KEN, 6KEO, 6KEP, 6KEQ, and 6KER. All other raw data are to be shared upon request to R.N. (ryusuke-niwa@tara.tsukuba.ac.jp).

## Author contributions

K. Koiwai, K. I., T. S., and R. N. conceptualization; K. Koiwai, K. I., K. M., R. Y., T. H., and T. S. data curation; K. Koiwai, K. I., K. M., S. E., R. Y., T. H., and R. N. formal analysis; K. Koiwai, K. I., K. M., S. E., R. Y., K. F., Y. S.-N., T. S., and R. N. validation; K. Koiwai, K. I., K. M., S. E., R. A., R. Y., T. H., K. Kato, K. F., Y. S.-N., A. N., and R. N. investigation; K. Koiwai, K. I., R. Y., K. F., and Y. S.-N. visualization; K. Koiwai, K. I., R. Y., K. F., T. S., and R. N. writing-original draft; K. Koiwai, K. I., T. S., and R. N. writing-review and editing; H. K., T. O., Y. F., H. I., F. Y., and T. S. resources; H. K., T. O., Y. F., H. I., R. Y., T. H., K. Kato, K. F., A. N., F. Y., and T. S. methodology; H. K., T. O., F. Y., and T. S. project administration; F. Y., T. S., and R. N. supervision; R. N. funding acquisition.

## Supplementary Material

Supporting Information

## Footnotes

5 The abbreviations used are:

20E: 20-hydroxyecdysone
GSTE: GST, EC 2.5.1.18
EST: 17β-estradiol
MD: molecular dynamics
G-site: GSH-binding site
H-site: hydrophobic substrate-binding pocket
RMSD: root mean square deviation
FMO: fragment molecular orbital
IFIE: interfragment interaction energy
ES: electrostatic energy
EX: exchange-repulsion energy
CT+mix: charge-transfer energy and higher-order mixed term
DI: dispersion energy
ns: nanoseconds
ERα: estrogen receptor alpha
CDS: coding sequence.
