# Supplementary material for: An integrated approach to unravel a crucial structural property required for the function of the insect steroidogenic Halloween protein Noppera-bo
Source: J Biol Chem. 2020 Apr 2;295(20):7154–67. doi: 10.1074/jbc.RA119.011463 (PMC7242711; doi:10.1074/jbc.RA119.011463)
Supplement: Supporting Information [file supp_295_20_7154__index.html]

An integrated approach to unravel a crucial structural property required for the function of the insect steroidogenic Halloween protein Noppera-bo — A structural property of Drosophila Halloween GST Noppera-bo — An integrated approach to unravel a crucial structural property required for the function of the insect steroidogenic Halloween protein Noppera-bo — A structural property of Drosophila Halloween GST Noppera-bo — Supporting Information 

# An integrated approach to unravel a crucial structural property required for the function of the insect steroidogenic Halloween protein Noppera-bo

## Supporting Information

- Supporting Information (to be published online) - Figs. S1 to S12, Tables S1 to S5, Legends of Movie 1 and 2
- Supporting Information (to be published online) - Movie 1
- Supporting Information (to be published online) - Movie 2
