# Supplementary material for: An integrated approach to unravel a crucial structural property required for the function of the insect steroidogenic Halloween protein Noppera-bo
Source: J Biol Chem. 2020 Apr 2;295(20):7154–67. doi: 10.1074/jbc.RA119.011463 (PMC7242711; doi:10.1074/jbc.RA119.011463)
Supplement: Supporting Information [file supp_RA119.011463_156438_3_supp_498023_q7y5mk.pdf]

Supplementary Information for

An integrated approach unravels a crucial structural property for the function of the insect steroidogenic Halloween protein Noppera-bo

Kotaro Koiwai, Kazue Inaba, Kana Morohashi, Sora Enya, Reina Arai, Hirotatsu Kojima, Takayoshi Okabe, Yuuta Fujikawa, Hideshi Inoue, Ryunosuke Yoshino, Takatsugu Hirokawa, Koichiro Kato, Kaori Fukuzawa, Yuko Shimada-Niwa, Akira Nakamura, Fumiaki Yumoto, Toshiya Senda and Ryusuke Niwa

Corresponding author: Ryusuke Niwa
Email: ryusuke-niwa@tara.tsukuba.ac.jp

This PDF file includes:

Figs. S1 to S12
Tables S1 to S5
Legends of Movie 1 and 2

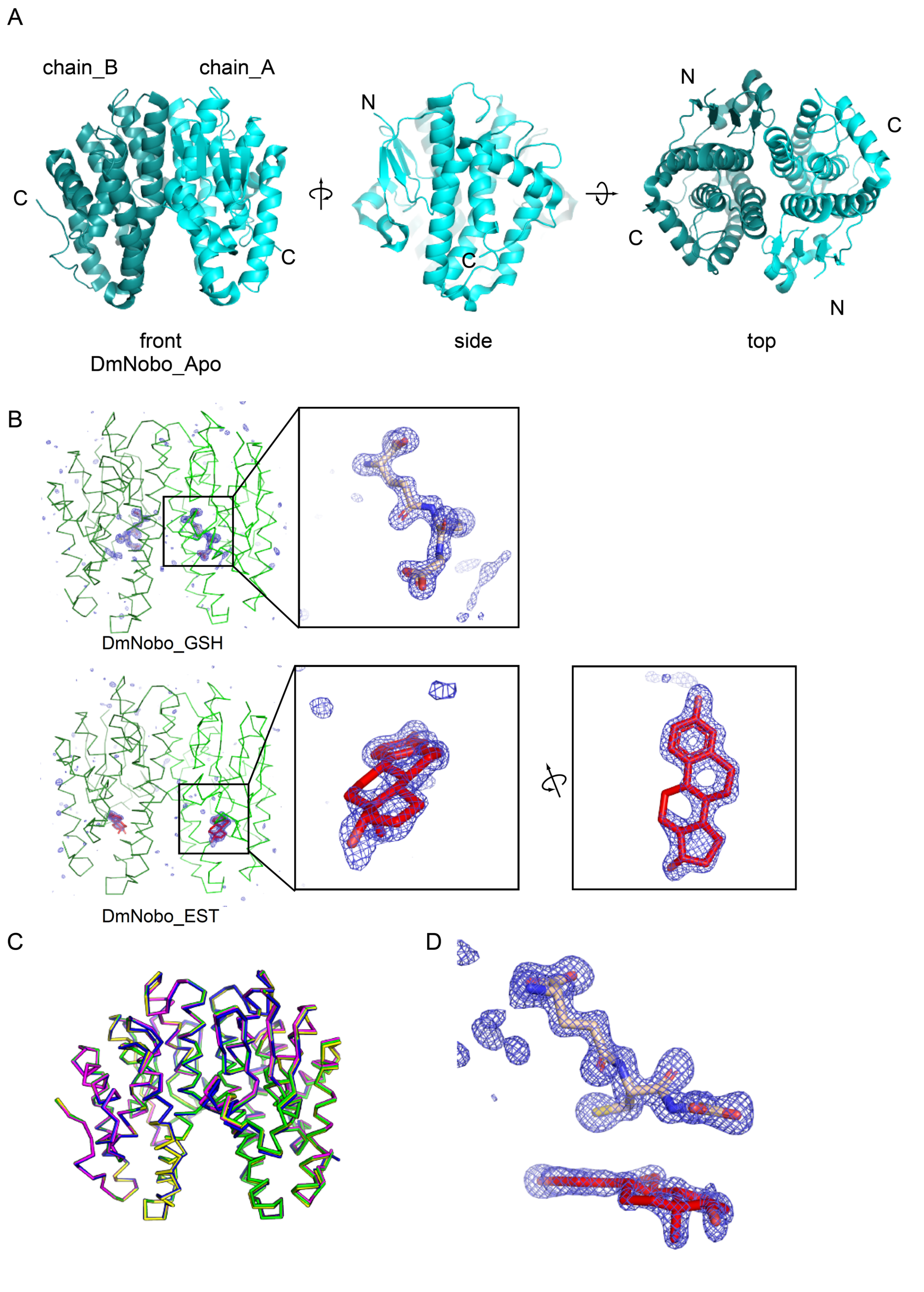

Fig. S1. Overall structures of DmNobo.

(A) The overall structure of DmNobo_Apo is represented as a ribbon diagram. Chains A and B are colored in cyan and turquoise, respectively. (B) Simulated annealing-omit maps of GSH and EST in DmNobo_GSH and DmNobo_EST, respectively. m*F*o-D*F*c maps (blue) are contoured at the 4.0σ level and shown with a blue mesh. A ribbon model of the DmNobo protein (green) and stick models of GSH and EST molecules are shown. Carbon atoms in GSH and EST are colored wheat and red, respectively. Oxygen atoms and nitrogen atoms are colored in green and blue, respectively. (C) Superimposed crystal structures of DmNobo_Apo

(blue), DmNobo_GSH (yellow), DmNobo_EST (magenta), and DmNobo_EST-GSH (green). Least square (LSQ) fittings were performed with the Cα atoms of chain A. RMSD values from the LSQ fittings are summarized in Table S1. (D) Simulated annealing-omit maps of GSH and EST in the DmNobo_EST-GSH structure. m*F*o-D*F*c maps (blue) are contoured at 4.0σ and shown with a blue mesh. GSH and EST molecules are shown as stick models.

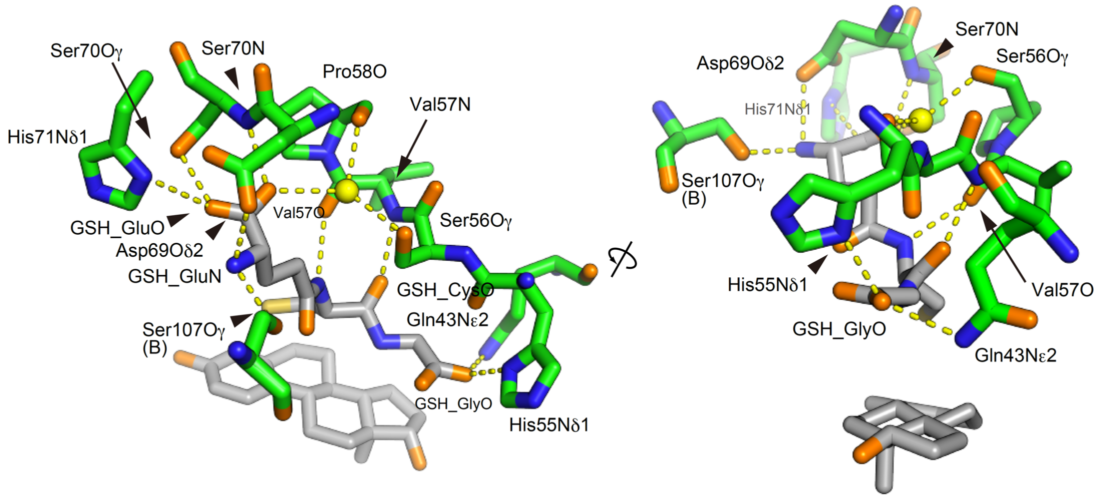

Fig. S2. A hydrogen-bond network between DmNobo and GSH.

Hydrogen bonds are indicated with yellow dashed lines. Carbon atoms of the protein and ligands (GSH, EST) are shown in green and gray, respectively. One of the two conformations of Ser107, in which the Oγ atom is directed towards GSH, is shown.

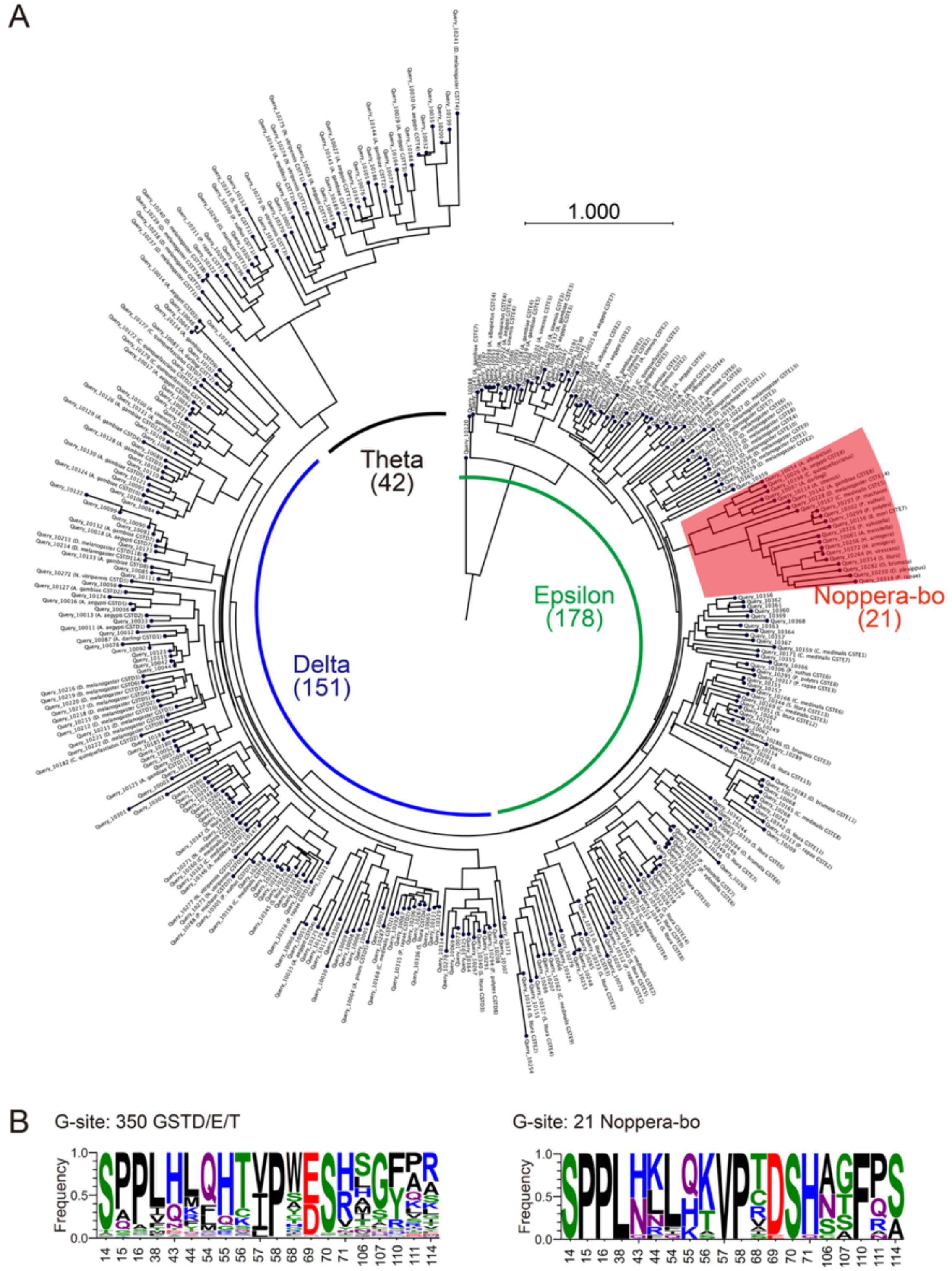

Fig. S3. Phylogenetic analysis of the delta, epsilon, and theta classes of GST.
(A) Phylogenetic tree of the insect GSTD/E/T proteins listed in Table S5. A clade of the *nobo* family is highlighted in red. (B) Frequencies of amino acid residues composing the G-site of 350 GSTD/E/T (left) and 21 Nobo (right) proteins displayed in A. The frequencies were calculated using LOGO, and Nobo proteins were excluded from the GSTD/E/T proteins for the frequency calculation.

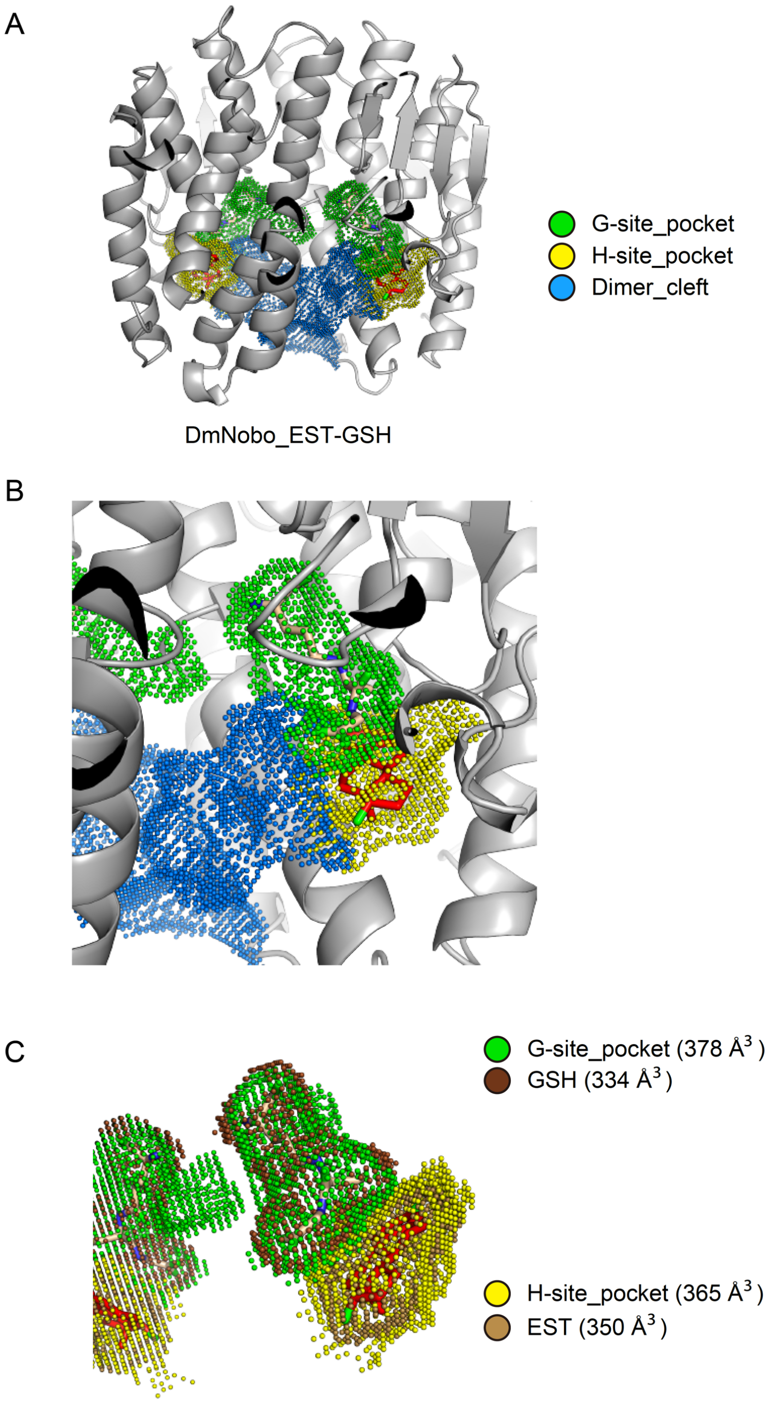

Fig. S4. Pockets in the G- and H-sites of DmNobo

(A, B) Pockets in DmNobo were calculated using 3V. The inner surfaces of the two pockets, i.e., the G- and H-sites, are represented in green and yellow dots, respectively. The cleft between the two subunits of the DmNobo_EST-GSH are shown in blue. The G-site was calculated using the crystal structure of the DmNobo_EST-GSH complex without GSH, and the H-site was calculated using the crystal structure of the DmNobo_EST-GSH complex without EST. (B) An enlarged view of the G- and H-sites. (C) The solvent-accessible surfaces of GSH and EST are represented with brown and light brown dots, respectively. The surfaces of the G- and H-sites are superimposed on GSH (green) and EST (yellow).

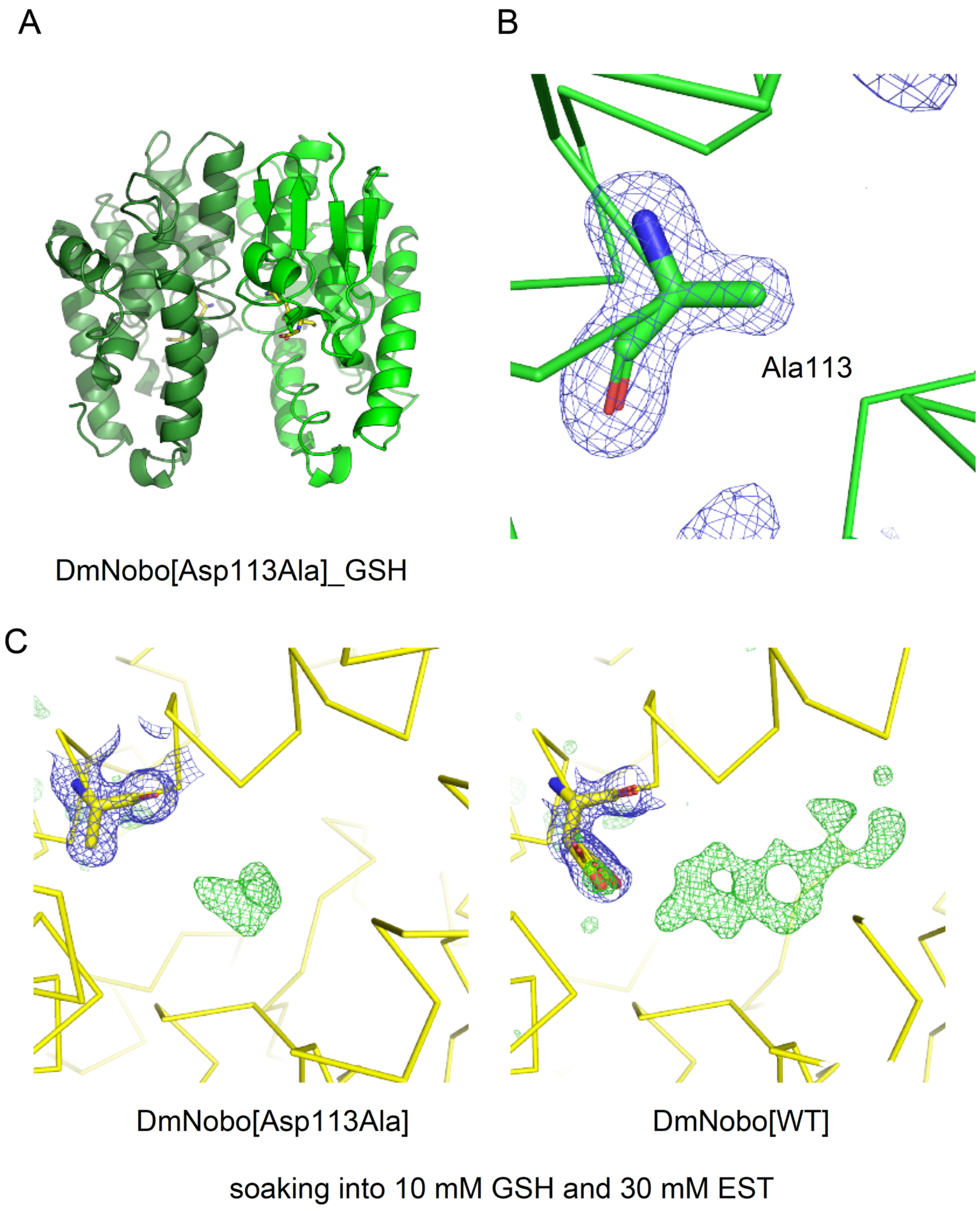

Fig. S5. Characterization of the DmNobo[Asp113Ala] protein.

(A) Overall structure of GSH in complex with DmNobo[Asp113Ala] (DmNobo[Asp113Ala]_GSH). Chains A and B are shaded green and dark green, respectively. The RMSD value from the LSQ fittings between Cα atoms in chain A of DmNobo[WT]_GSH and those of DmNobo[Asp113Ala] was 0.1 Å.

(B) Simulated annealing-omit maps of Ala113 of DmNobo[Asp113Ala]_GSH. m*F*o-D*F*c map (blue) contoured at 4.0σ was overlaid with sticks of Ala113 and a ribbon model of DmNobo[Asp113Ala]_GSH (green). (C) m*F*o-D*F*c map around the H-sites of DmNobo[Asp113Ala] and DmNobo[WT]. EST did not bind to DmNobo[Asp113Ala]. Crystals of DmNobo[Asp113Ala] and DmNobo[WT] were soaked into an artificial mother liquor (42.5% [w/v] PPG 400 in 100 mM Bis-Tris [pH 6.4]) containing 10 mM GSH and 30 mM EST.

The m*F*o-D*F*c maps contoured at 4.0σ (green mesh) and the 2m*F*o-D*F*c map contoured at 1.5σ around residue 113 (within 5 Å from residue 113, blue mesh) are shown.

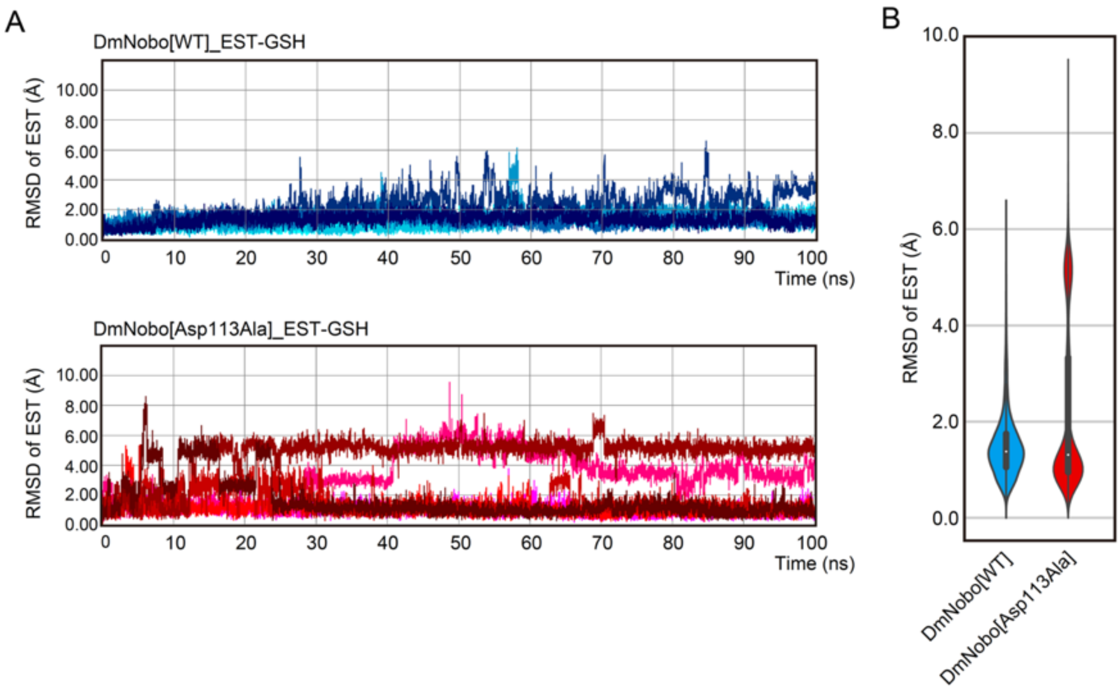

Fig. S6. *In silico* evaluation of the contribution of Asp113 to the interaction between DmNobo and EST.

(A) The RMSD values of EST in DmNobo[WT] (top) or DmNobo[Asp113Ala] (bottom) in triplicate independent calculations. All protein frames were aligned based on the protein backbone of the initial structure. The RMSD (Å) of EST between EST at the initial frame and each subsequent frame is plotted. (B) Violin-plot of the RMSD of EST in DmNobo[WT] or DmNobo[Asp113Ala]. The median, interquartile range, and 95% confidence interval are shown with a white dot, a bold line, and a narrow line, respectively. The width of the plot indicates the frequency of the frames.

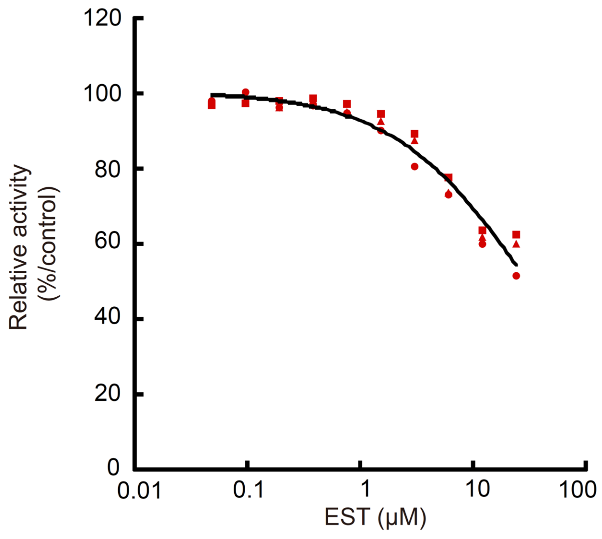

Fig. S7. EST-dependent inhibition of the GSH-conjugation activities of AgNobo[Asp111Ala].

3,4-DNADCF was used as an artificial fluorescent substrate. Relative activity is defined as the ratio of activity when compared to the protein without EST. All of the data points in triplicate assays are indicated. The value of IC_{50} was >25 μM.

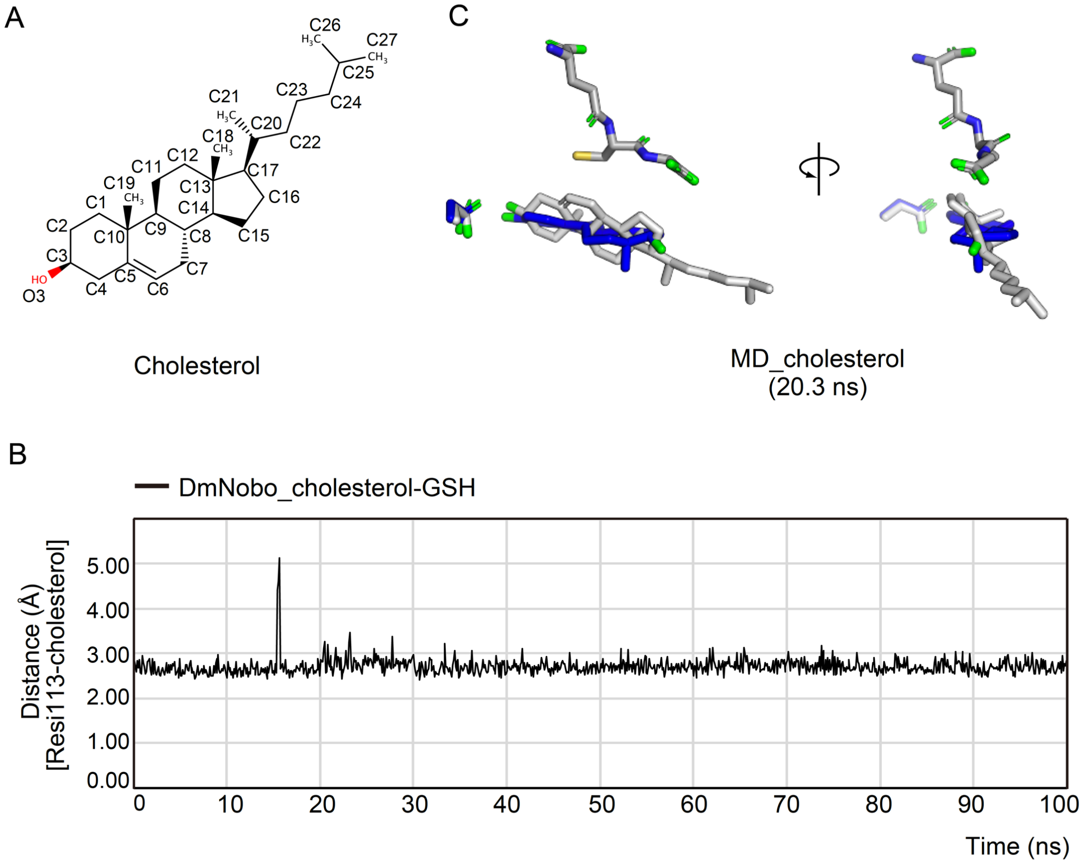

Fig. S8. ***In silico*** **evaluation of interaction between DmNobo and cholesterol**

(A) Chemical structure of cholesterol. (B) MD-simulation results for DmNobo in complex with cholesterol and GSH. The distance between Oδ of Asp113 of DmNobo and O3 of cholesterol was plotted against time. (C) MD models of DmNobo_cholesterol-GSH and DmNobo_EST-GSH at 20.3 ns.

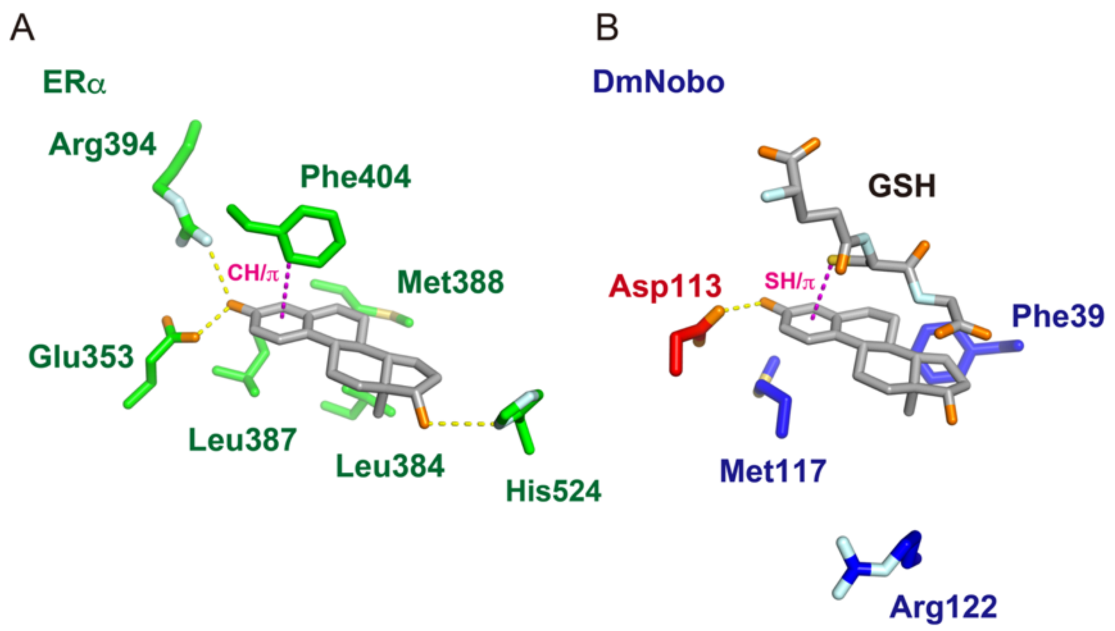

Fig. S9. Comparison of the DmNobo_EST and ERα_EST interactions.

Side chains of representative residues of ERα (A) or DmNobo (B) that interact with EST are represented with sticks (PDB ID for ERα structure = 1QKT). A hydrogen bond between the protein and EST is indicated with a yellow dashed line. A CH/π interaction between Phe404 and EST or an SH/π interaction between GSH and EST is indicated with a pink dashed line.

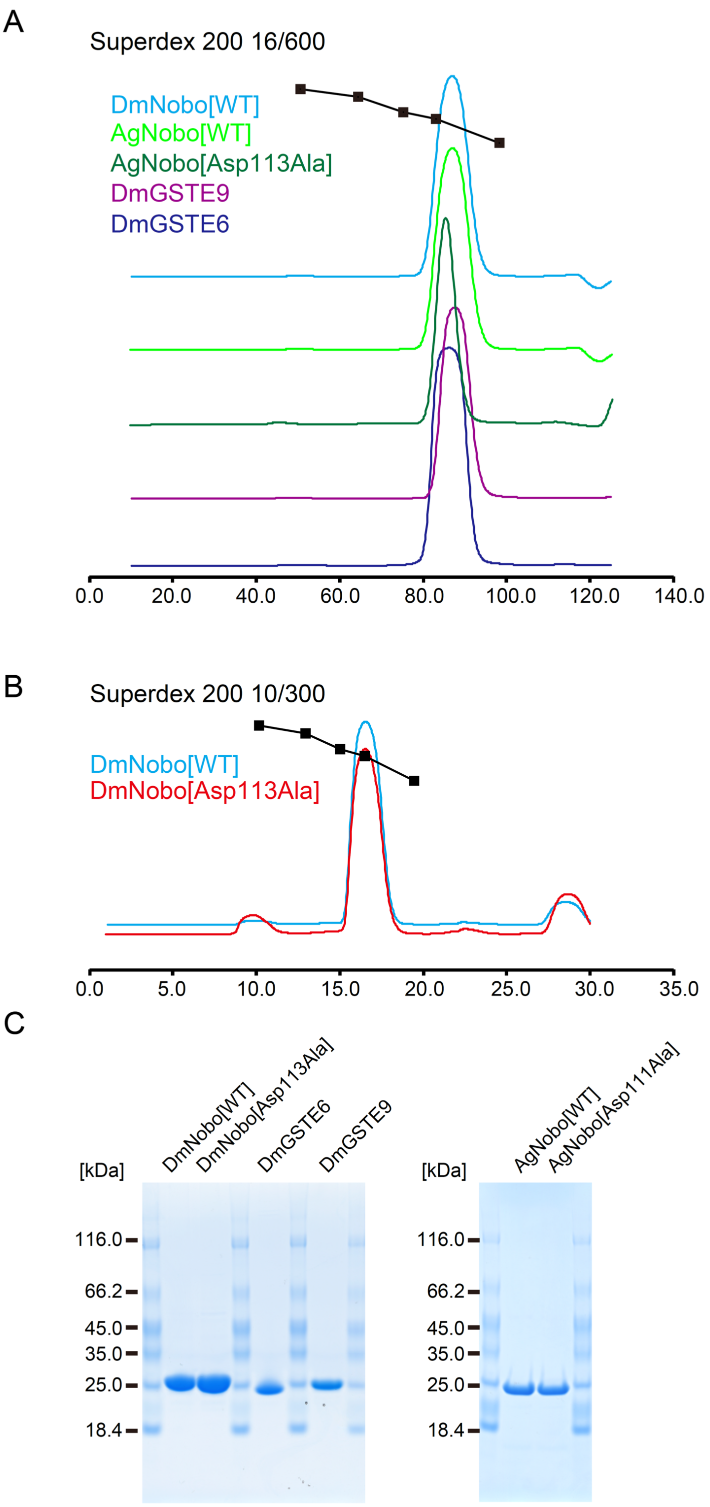

Fig. S10. Purified proteins in this study

(A) DmNobo[WT], DmGSTE6, DmGSTE9, AgNobo[WT] and AgNobo[Asp111Ala] were purified by size exclusion column chromatography with HiLoad Superdex 200 16/600 (GEhealthcare). UV charts of DmNobo[WT] (cyan), AgNobo[WT] (light green), AgNobo[Asp111Ala] (dark green), DmGSTE6 (blue), and DmGSTE9 (purple) are shown together with peak points of marker proteins. The marker proteins used were β-amylase from sweet potato (223.8 kDa), alcohol dehydrogenase from *Saccharomyces cerevisiae* (146.8

kDa), bovine serum albumin (66.5 kDa), human GSTP1-1 (46.7 kDa in solution), and RNase A (13.7 kDa).
(B) DmNobo[Asp113Ala] was purified by size exclusion column chromatography with Superdex 200 increase 10/300 (GEhealthcare). UV charts of DmNobo[Asp113Ala] (red) and DmNobo[WT] (cyan) as a control are shown together with peak points of the marker proteins.
(C) SDS-PAGE analyses for DmNobo[WT], DmNobo[Asp113Ala], DmGSTE6, DmGSTE9, AgNobo[WT] and AgNobo[Asp111Ala]. Purified proteins were fractionated by SDS-PAGE in reduced condition, and stained by Coomassie Brilliant Blue G-250.

GATGTTCGCCCTGGGCAGCAGTCTCAATTGGCGCGAGAGGTACATTGTTTAGCGG
GCTGGACACAAATCAACTTGATTTTCAGTAATAAAACCAAGAAAATTAAATGTTT
TTCTGGTTTAAGTGGGGAAAAATGCAGGTGCTTCTTCTTGTCCAGAAAGTGTGGC
CGATCAGCTGAGTCTAACAGTCCGGTTAACAGTGCGTTATAGTATGTCGTTTTCCA
GCACTACATCAGGGATACCACACAAATTGAAAAATGAGCGCCAAATTTAAAATG
ACTTCGTTATCTATCTTATCAATTTCAATTTCCATAGATCTGTACTTTTGGTGAAA
GTGAACAGTGTTTATATATTTGATTTAATATAACAATTAATTGATGAATTATCTC
GTAGTATACATTAAAATGCCACCATCAATCAGTACTGTGGCTACAAATGCTTTTC
AATTCAGTTACTGATCGACTTTCAAGGCGTTCGTTTGTCTGTTTTTCTACCTGATCC
TAACTTAATGGATTACAAGGATCACGATGGCGACTACAAGGACCATGACATCGA
CTATAAGGACGACGACGATAAGGGATCCTATCCATATGACGTTCCAGATTACGCT
ATGTCTCAGCCCAAGCCGATTTTGTATTATGATGAGCGCAGCCCACCAGTCCGCA
GTTGCCTTATGCTAATCAAATTGCTCGATATAGATGTGGAGCTCAGGTTTGTGAAT
CTCTTCAAGGGCGAGCAATTCCAAAAAGATTTCTTAGCGGTAAGTAAGTCAAATT
TATTCAATGAATAAATTGAATCAATCATTATTTAAGAAAACGAATAATTTAATTT
GCGAAAATTCCTTATAATTATAATTTCTGTTTTATATTTTGCCATTTTTATTTAGTT
AAATCCCCAGCACAGTGTTCCCACCCTAGTCCACGGTGATCTGGTGCTGACGGAC
AGTCATGCTATACTCATTCACCTGGCGGAGAAGTTCGATGAGGGCGGTAGTTTGT
GGCCGCAGGAGCACGCAGAACGGATGAAGGTTCTGAACCTCCTGCTCTTCGAGTG
CTCCTTTTTGTTCCGACGTGACAGTGATTTTATGGTGGGTGATTCGCAACTAAAAC
TAGCTGTTTACATATTTTGTTTTTTATTTTTCCCAGTCGGCGACTGGGCGCTCTTCC
GCTTCCTCGCTCACTGACTCGCTGCGCTCGGTCGTTCGGCTGCGGCGAGCGGTATC
AGCTCACTCAAAGGCGGTAATACGGTTATCCACAGAATCAGGGGATAACGCAGG
AAAGAACATGTGAGCAAAAGGCCAGCAAAAGGCCAGGAACCGTAAAAAGGCCG
CGTTGCTGGCGTTTTTCCATAGGCTCCGCCCCCCTGACGAGCATCACAAAAATCG
ACGCTCAAGTCAGAGGTGGCGAAACCCGACAGGACTATAAAGATACCAGGCGTT
TCCCCCTGGAAGCTCCCTCGTGCGCTCTCCTGTTCCGACCCTGCCGCTTACCGGAT
ACCTGTCCGCCTTTCTCCCTTCGGGAAGCGTGGCGCTTTCTCATAGCTCACGCTGT
AGGTATCTCAGTTCGGTGTAGGTCGTTCGCTCCAAGCTGGGCTGTGTGCACGAAC
CCCCCGTTCAGCCCGACCGCTGCGCCTTATCCGGTAACTATCGTCTTGAGTCCAAC
CCGGTAAGACACGACTTATCGCCACTGGCAGCAGCCACTGGTAACAGGATTAGC
AGAGCGAGGTATGTAGGCGGTGCTACAGAGTTCTTGAAGTGGTGGCCTAACTACG
GCTACACTAGAAGAACAGTATTTGGTATCTGCGCTCTGCTGAAGCCAGTTACCTT
CGGAAAAAGAGTTGGTAGCTCTTGATCCGGCAAACAAACCACCGCTGGTAGCGG
TGGTTTTTTTGTTTGCAAGCAGCAGATTACGCGCAGAAAAAAAGGATCTCAAGAA
GATCCTTTGATCTTTTCTACGGGGTCTGACGCTCAGTGGAACGAAAACTCACGTT
AAGGGATTTTGGTCATGAGATTATCAAAAAGGATCTTCACCTAGATCCTTTTAAA
TTAAAAATGAAGTTTTAAATCAATCTAAAGTATATATGAGTAAACTTGGTCTGAC
AGTTACCAATGCTTAATCAGTGAGGCACCTATCTCAGCGATCTGTCTATTTCGTTC
ATCCATAGTTGCCTGACTCCCCGTCGTGTAGATAACTACGATACGGGAGGGCTTA
CCATCTGGCCCCAGTGCTGCAATGATACCGCGAGATCCACGCTCACCGGCTCCAG
ATTTATCAGCAATAAACCAGCCAGCCGGAAGGGCCGAGCGCAGAAGTGGTCCTG
CAACTTTATCCGCCTCCATCCAGTCTATTAATTGTTGCCGGGAAGCTAGAGTAAGT
AGTTCGCCAGTTAATAGTTTGCGCAACGTTGTTGCCATTGCTACAGGCATCGTGGT
GTCACGCTCGTCGTTTGGTATGGCTTCATTCAGCTCCGGTTCCCAACGATCAAGGC
GAGTTACATGATCCCCCATGTTGTGCAAAAAAGCGGTTAGCTCCTTCGGTCCTCC
GATCGTTGTCAGAAGTAAGTTGGCCGCAGTGTTATCACTCATGGTTATGGCAGCA
CTGCATAATTCTCTTACTGTCATGCCATCCGTAAGATGCTTTTCTGTGACTGGTGA
GTACTCAACCAAGTCATTCTGAGAATAGTGTATGCGGCGACCGAGTTGCTCTTGC
CCGGCGTCAATACGGGATAATACCGCGCCACATAGCAGAACTTTAAAAGTGCTCA
TCATTGGAAAACGTTCTTCGGGGCGAAAACTCTCAAGGATCTTACCGCTGTTGAG
ATCCAGTTCGATGTAACCCACTCGTGCACCCAACTGATCTTCAGCATCTTTTACTT
TCACCAGCGTTTCTGGGTGAGCAAAAACAGGAAGGCAAAATGCCGCAAAAAAGG
GAATAAGGGCGACACGGAAATGTTGAATACTCATACTCTTCCTTTTTCAATATTAT
TGAAGCATTTATCAGGGTTATTGTCTCATGAGCGGATACATATTTGAATGTATTTA

GAAAAATAAACAAATAGGGGTTCCGCGCACATTTCCCCGAAAAGTGCCACCTGA
CGTCTAAGAAACCATTATTATCATGACATTAACCTATAAAAATAGGCGTATCACG
AGGCCCTTTCGTC

Fig. S11.
DNA sequence of pDonor[KI]-{CG4688_LA}:{3×FLAG/HA/nobo}:{CG4688_RA}
Gray highlighting: 5′ homology arm; blue highlighting: ATG + 3× FLAG tag; yellow highlighting: HA tag; magenta highlighting: exons of Nobo (CG4688); green highlighting: introns of Nobo (CG4688); underlined text: pUC19 ori

GATGTTCGCCCTGGGCAGCAGTCTCAATTGGCGCGAGAGGTACATTGTTTAGCGG
GCTGGACACAAATCAACTTGATTTCAGTAATAAAACCAAGAAAATTAAATGTTT
TTCTGGTTTAAGTGGGGAAAAATGCAGGTGCTTCTTCTTGTCCAGAAAGTGTGGC
CGATCAGCTGAGTCTAACAGTCCGGTTAACAGTGCGTTATAGTATGTCGTTTTCCA
GCACTACATCAGGGATACCACACAAATTGAAAAATGAGCGCCAAATTTAAAATG
ACTTCGTTATCTATCTTATCAATTTCAATTTCCATAGATCTGTACTTTTGGTGAAA
GTGAACAGTGTTTATATATTTGATTTTAATATAACAATTAATTGATGAATTATCTC
GTAGTATACATTAAAATGCCACCATCAATCAGTACTGTGGCTACAAATGCTTTTC
AATTCAGTTACTGATCGACTTTCAAGGCGTTCGTTTGTCTGTTTTTCTACCTGATCC
TAACTTAATGGATTACAAGGATCACGATGGCGACTACAAGGACCATGACATCGA
CTATAAGGACGACGACGATAAGGGATCCTATCCATATGACGTTCCAGATTACGCT
ATGTCTCAGCCCAAGCCGATTTTGTATTATGATGAGCGCAGCCCACCAGTCCGCA
GTTGCCTTATGCTAATCAAATTGCTCGATATAGATGTGGAGCTCAGGTTTGTGAAT
CTCTTCAAGGGCGAGCAATTCCAAAAAGATTTCTTAGCGGTAAGTAAGTCAAATT
TATTCAATGAATAAATTGAATCAATCATTATTTTAAGAAAACGAATAATTTAATTT
GCGAAAATTCCTTATAATTATAATTTCTGTTTTATATTTTGCCATTTTTATTTAGTT
AAATCCCCAGCACAGTGTTCCCACCCTAGTCCACGGTGATCTGGTGCTGACGGAC
AGTCATGCTATACTCATTCACCTGGCGGAGAAGTTCGATGAGGGCGGTAGTTTGT
GGCCGCAGGAGCACGCAGAACGGATGAAGGTTCTGAACCTCCTGCTCTTCGAGTG
CTCCTTTTTGTTCCGACGT**GCC**AGCGACTTCATGGTGGGTGATTCGCAACTAAAA
CTAGCTGTTTACATATTTTGTTTTTTATTTTTCCCAGTCGGCGACTGTCCGCCAGGG
ATTCGCCAATGTCGATGTGGCACATCATGAACGCAAGCTGACCGAGGCGTATATT
ATCATGGAGCGTTACCTGGAAAATAGCGATTTTATGGCCGGGCCACAGGTAAAA
AAAGACCAGCTCATCTGATTGCGATTCCCTGTCGTTGTGGCGGCTCAATTAAGTCT
AGAATTTCACCTTTGTTTCCGCGGTTTCAGCTGACGCTCGCCGACTTATCCATCGT
GACCACATTGAGCACCGTCAATCTCATGTTTCCCCTGTCGCAGTTCCCACGTCTGC
GGCGCTGGTTCACCGCGATGCAGCAGCTGGATGCCTACGAGGCCAACTGCAGTG
GCTTGGAGAAGCTCCGCCAAACGATGGAGAGCGTCGGTAGCTTTCAGTTCCCATC
GTCATCAGCGGTAGTCACCGAGAAGGTGGAGTAGGGCGCTCTTCCGCTTCCTCGC
TCACTGACTCGCTGCGCTCGGTCGTTCGGCTGCGGCGAGCGGTATCAGCTCACTC
AAAGGCGGTAATACGGTTATCCACAGAATCAGGGGATAACGCAGGAAAGAACAT
GTGAGCAAAAGGCCAGCAAAAGGCCAGGAACCGTAAAAAGGCCGCGTTGCTGGC
GTTTTTCCATAGGCTCCGCCCCCCTGACGAGCATCACAAAAATCGACGCTCAAGT
CAGAGGTGGCGAAACCCGACAGGACTATAAAGATACCAGGCGTTTCCCCCTGGA
AGCTCCCTCGTGCGCTCTCCTGTTCCGACCCTGCCGCTTACCGGATACCTGTCCGC
CTTTCTCCCTTCGGGAAGCGTGGCGCTTTCTCATAGCTCACGCTGTAGGTATCTCA
GTTCGGTGTAGGTCGTTCGCTCCAAGCTGGGCTGTGTGCACGAACCCCCCGTTCA
GCCCGACCGCTGCGCCTTATCCGGTAACTATCGTCTTGAGTCCAACCCGGTAAGA
CACGACTTATCGCCACTGGCAGCAGCCACTGGTAACAGGATTAGCAGAGCGAGG
TATGTAGGCGGTGCTACAGAGTTCTTGAAGTGGTGGCCTAACTACGGCTACACTA
GAAGAACAGTATTTGGTATCTGCGCTCTGCTGAAGCCAGTTACCTTCGGAAAAAG
AGTTGGTAGCTCTTGATCCGGCAAACAAACCACCGCTGGTAGCGGTGGTTTTTTT
GTTTGCAAGCAGCAGATTACGCGCAGAAAAAAAGGATCTCAAGAAGATCCTTTG
ATCTTTTCTACGGGGTCTGACGCTCAGTGGAACGAAAACTCACGTTAAGGGATTT
TGGTCATGAGATTATCAAAAAGGATCTTCACCTAGATCCTTTTAAATTAAAAATG
AAGTTTTAAATCAATCTAAAGTATATATGAGTAAACTTGGTCTGACAGTTACCAA
TGCTTAATCAGTGAGGCACCTATCTCAGCGATCTGTCTATTTCGTTCATCCATAGT
TGCCTGACTCCCCGTCGTGTAGATAACTACGATACGGGAGGGCTTACCATCTGGC
CCCAGTGCTGCAATGATACCGCGAGATCCACGCTCACCGGCTCCAGATTTATCAG
CAATAAACCAGCCAGCCGGAAGGGCCGAGCGCAGAAGTGGTCCTGCAACTTTAT
CCGCCTCCATCCAGTCTATTAATTGTTGCCGGGAAGCTAGAGTAAGTAGTTCGCC
AGTTAATAGTTTGCGCAACGTTGTTGCCATTGCTACAGGCATCGTGGTGTCACGCT
CGTCGTTTGGTATGGCTTCATTCAGCTCCGGTTCCCAACGATCAAGGCGAGTTAC
ATGATCCCCCATGTTGTGCAAAAAAGCGGTTAGCTCCTTCGGTCCTCCGATCGTTG
TCAGAAGTAAGTTGGCCGCAGTGTTATCACTCATGGTTATGGCAGCACTGCATAA

TTCTCTTACTGTCATGCCATCCGTAAGATGCTTTTCTGTGACTGGTGAGTACTCAA
CCAAGTCATTCTGAGAATAGTGTATGCGGCGACCGAGTTGCTCTTGCCCGGCGTC
AATACGGGATAATACCGCGCCACATAGCAGAACTTTAAAAGTGCTCATCATTGGA
AAACGTTCTTCGGGGCGAAAACTCTCAAGGATCTTACCGCTGTTGAGATCCAGTT
CGATGTAACCCACTCGTGCACCCAACTGATCTTCAGCATCTTTTACTTTCACCAGC
GTTTCTGGGTGAGCAAAAACAGGAAGGCAAAATGCCGCAAAAAAGGGAATAAG
GGCGACACGGAAATGTTGAATACTCATACTCTTCCTTTTTCAATATTATTGAAGCA
TTTATCAGGGTTATTGTCTCATGAGCGGATACATATTTGAATGTATTTAGAAAAAT
AAACAAATAGGGGTTCCGCGCACATTTCCCCGAAAAGTGCCACCTGACGTCTAAG
AAACCATTATTATCATGACATTAACCTATAAAAATAGGCGTATCACGAGGCCCTT
TCGTC

Fig. S12.
DNA sequence of pDonor[KI]-{CG4688_LA}:{3×FLAG/HA/nobo*D113A}:{CG4688_RA}
Gray highlighting: 5′ homology arm; blue highlighting: ATG + 3× FLAG tag; yellow highlighting: HA tag; magenta highlighting: exons of Nobo (CG4688); green highlighting: introns of Nobo (CG4688); boxed and bold text: substitution with Ala (GAC → GCC); underlined text: pUC19 ori

Table S1. Crystallographic Summary of DmNobo crystal structures

	DmNobo_Apo	DmNobo_GSH	DmNobo_EST	DmNobo_EST-GSH
Data Collection				
Space group	$P2_12_12_1$	$P2_12_12_1$	$P2_12_12_1$	$P2_12_12_1$
Cell Dimensions				
a, *b*, *c* (Å)	59.12, 76.56 106.36	58.75, 75.67, 107.84	58.40, 75.08, 109.02	58.38, 75.06, 108.52
α, β, γ (°)	90.00, 90.00, 90.00	90.00, 90.00, 90.00	90.00, 90.00, 90.00	90.00, 90.00, 90.00
Resolution* (Å)	42.83 - 1.50 (1.55 - 1.50)	46.42 - 1.75 (1.81 - 1.75)	46.12 - 1.58 (1.64 - 1.58)	46.11 - 1.55 (1.61 - 1.55)
R_{merge}	0.045 (0.38)	0.082 (1.05)	0.048 (0.84)	0.038 (0.36)
I/σI	33.8 (6.53)	27.3 (2.3)	37.6 (2.6)	42.7 (3.3)
Completeness	1.00 (1.00)	1.00 (0.97)	0.87 (0.47)	0.95 (0.69)
Redundancy	13.1 (12.7)	14.3 (12.2)	14.1 (10.6)	12.3 (3.8)
CC1/2	1.00 (0.97)	1.00 (0.75)	1.00 (0.76)	1.00 (0.84)
Refinement				
Resolution (Å)	42.83 - 1.50	35.70 - 1.75	35.49 - 1.70	32.59 - 1.55
No. of reflections	77,842	49,095	51,170	65,906
R_{work}	0.164	0.173	0.176	0.161
R_{free}	0.195	0.207	0.211	0.182
No. of Atoms				
Protein	3,880	3,609	3,687	3,817
GSH	0	40	0	40
EST	0	0	40	40
Water	643	408	403	499
B factors				
Protein	22.0	22.0	23.5	20.1
GSH	-	17.7	-	15.5
EST	-	-	31.5	20.8
Waters	39.5	33.7	34.5	34.7
RMSD				
Bond length (Å)	0.008	0.007	0.008	0.010
Bond angles (°)	0.98	0.89	0.96	1.19
Ramachandran plot (%)				
favored	99.1	99.3	98.9	99.3
allowed	0.90	0.68	1.14	0.68
outliers	0.00	0.00	0.00	0.00
PDB ID	6KEM	6KEN	6KEO	6KEP

Each structure was determined from diffraction data from one crystal. PDB, Protein Data Bank; RMSD, root-mean-square deviation. *Highest resolution shells are shown in parentheses.

Table S1. Crystallographic Summary of DmNobo crystal structures *Continued*

	DmNobo[Asp113Ala]_Apo	DmNobo[Asp113Ala]_GSH
Data Collection		
Space group	$P2_12_12_1$	$P2_12_12_1$
Cell Dimensions		
a, *b*, *c* (Å)	58.72, 75.48, 107.11	58.37, 74.83, 107.42
α, β, γ (°)	90.00, 90.00, 90.00	90.00, 90.00, 90.00
Resolution* (Å)	46.34 - 1.84 (1.90 - 1.84)	46.03 - 1.84 (1.90 - 1.84)
R_{merge}	0.072 (1.10)	0.113 (1.12)
I/σI	21.3 (2.1)	15.2 (2.12)
Completeness	1.00 (0.99)	99.9 (99.5)
Redundancy	13.3 (13.8)	13.5 (13.9)
CC1/2	1.00 (0.83)	1.00 (0.78)
Refinement		
Resolution (Å)	46.34 - 1.84	46.03 - 1.84
No. reflections	42,099	41,768
R_{work}	0.208	0.189
R_{free}	0.240	0.221
No. of Atoms		
Protein	3,495	3,529
GSH	0	40
EST	0	0
Water	174	146
B factors		
Protein	33.51	21.9
GSH	-	18.1
EST	-	-
Waters	36.85	26.5
RMSD		
Bond length (Å)	0.005	0.004
Bond angles (°)	0.73	0.66
Ramachandran plot (%)		
favored	99.0	99.1
allowed	0.91	0.91
outliers	0.00	0.00
PDB ID	6KEQ	6KER

Table S2. root-mean-square deviation (RMSD) among DmNobo crystal structures

		RMSD (Å) of Cα atoms of chain A[†]/ RMSD (Å) of Cα atoms of chains A and B[‡]		
	Coordinate error (Å)[*]	DmNobo_GSH	DmNobo_EST	DmNobo_EST-GSH
DmNobo_Apo	0.13	0.20 / 0.35	0.26 / 0.42	0.48 / 0.55
DmNobo_GSH	0.20		0.16 / 0.24	0.42 / 0.36
DmNobo_EST	0.21			0.40 / 0.32
DmNobo_EST-GSH	0.15			

[*] Coordinate errors are estimated by a maximum-likelihood method.

[†] Number of aligned Cα atoms in chain A: 199 atoms

[‡] Number of aligned Cα atoms in chain A and B: 391 atoms

Table S3. Summary of H-site-composing, or EST-interacting atoms in DmNobo

* Atoms of DmNobo within 4.0 Å from EST.
† Atoms of EST within 4.0 Å from each EST-interacting atom.

Residue	H-site-composing atoms	EST-interacting atoms*	DmNobo-interacting atoms†	Distance ‡ (Å)	Identity among DmGST D/E/T	Identity among Nobo	Total IFIE to EST (kcal/mol)
Arg13	C, Cβ	-	-	-	0.07	0.05	-0.31
Ser14	Cα, N, Cβ	-	-	-	0.85	1.00	-1.78
Pro15	Cδ, Cγ	Cδ	O3	3.2	0.62	1.00	-3.93
		Cδ	C3	3.6			
		Cδ	C4	3.7			
		Cγ	O3	3.1			
Leu38	C, O, Cβ, Cδ	Cβ	C15	4.0	0.61	1.00	-1.86
		Cδ	C7	4.0			
Phe39	Cα, Cδ, Cγ, Cε, Cζ	Cε1	C6	3.9	0.07	1.00	-6.77
			C7	3.7			
		π	C15	3.9			
Gln43	Nε	-	-	-	0.21	0.05	-0.51
Phe110	Cε, Cζ	Cε	C2	4.0	0.42	1.00	-2.79
Asp113	Oδ	Oδ	O3	2.6	0.01	0.95	-41.4
Ser114	Cα, Cβ	Cα	C1	3.8	0.10	0.76	-2.17
		Cα	C2	3.8			
		Cβ	C1	3.9			
Met117	Cβ, Cγ, Cε	Cβ	C1	3.9	0.04	0.40	-3.52
		Cβ	C2	3.9			
		Cγ	C2	3.8			
		Cγ	C3	3.9			
Ser118	Cα, N, Cβ, Cγ	-	-	-	0.06	0.33	-3.25
Val121	Cβ, Cγ1, Cγ2	Cβ	C18	3.9	0.11	0.05	-1.63
		Cγ1	C18	3.9			
		Cγ2	C18	3.8			
Arg122	Cβ	-	-	-	0.07	0.14	-1.49
Thr172	Cγ	Cγ	O3	3.3	0.31	0.52	0.026
Leu208	Cδ1, Cδ2	Cδ1	C4	3.9	0.31	0.95	-2.17
		Cδ1	C6	3.8			
		Cδ2	O3	4.0			
		Cδ2	C4	3.9			
Met212	Sδ	-	-	-	0.05	0.95	-0.65
GSH	Cys_Cβ, Cys_Sγ	Cys_Sγ	π	3.7	-	-	-3.48

‡ Average of chains A and B in the asymmetric unit.

Table S4. FMO analysis between fragments in DmNobo and EST

Residue	Total [kcal/mol]	ES [kcal/mol]	EX [kcal/mol]	CT [kcal/mol]	DI [kcal/mol]
Arg13	-0.31	0.36	0.03	-0.10	-0.59
Ser14	-1.78	0.49	0.20	-1.01	-1.47
Pro15	-3.93	-1.52	2.53	-1.25	-3.69
Arg18	-1.85	-1.66	0.00	-0.02	-0.17
Leu38	-1.86	-0.67	2.75	-0.79	-3.15
Phe39	-6.77	-1.77	4.34	-1.97	-7.37
Lys40	0.13	0.28	0.00	0.00	-0.15
Gln43	-0.51	0.25	0.12	-0.18	-0.69
Phe110	-2.79	-1.08	0.31	-0.51	-1.51
Asp113	-41.38	-49.71	26.87	-12.63	-5.91
Ser114	-2.17	-0.03	3.72	-2.76	-3.11
Asp115	3.31	1.66	0.07	2.18	-0.59
Met117	-3.52	0.12	2.12	-1.36	-4.39
Ser118	-3.25	-0.41	0.87	-1.05	-2.65
Ala119	-0.36	-0.24	0.03	0.24	-0.39
Val121	-1.63	-0.74	2.35	-0.47	-2.77
Arg122	-1.49	-1.10	0.00	-0.03	-0.36
Leu208	-2.17	-0.74	2.23	-0.70	-2.97
GSH_Gly	-2.49	0.64	0.32	-0.89	-2.56
GSH_Cys	-3.48	-1.00	5.12	-1.68	-5.92
PIEDA	-82.4	-59.2	55.8	-25.8	-53.1

Table S5. ID of amino acid sequences used in phylogenetic analysis

Accession	Description
Query_10001	tr|C4WSG3|C4WSG3_ACYPI ACYPI009122 protein OS=Acyrthosiphon pisum OX=7029 GN=ACYPI009122 PE=2 SV=1
Query_10002	tr|C4WT83|C4WT83_ACYPI ACYPI008657 protein OS=Acyrthosiphon pisum OX=7029 GN=ACYPI008657 PE=2 SV=1
Query_10003	tr|C4WUF9|C4WUF9_ACYPI ACYPI005620 protein OS=Acyrthosiphon pisum OX=7029 GN=ACYPI005620 PE=2 SV=1
Query_10004	tr|I6SCG3|I6SCG3_ACYPI Glutathione S-transferase D5 OS=Acyrthosiphon pisum OX=7029 GN=GstD5 PE=3 SV=1
Query_10005	tr|J9JLJ3|J9JLJ3_ACYPI Uncharacterized protein OS=Acyrthosiphon pisum OX=7029 PE=4 SV=2
Query_10006	tr|J9K2A8|J9K2A8_ACYPI Uncharacterized protein OS=Acyrthosiphon pisum OX=7029 PE=3 SV=1
Query_10007	tr|J9K372|J9K372_ACYPI Uncharacterized protein OS=Acyrthosiphon pisum OX=7029 PE=3 SV=1
Query_10008	tr|J9K6N7|J9K6N7_ACYPI Uncharacterized protein OS=Acyrthosiphon pisum OX=7029 GN=100167788 PE=4 SV=1
Query_10009	tr|J9M2I6|J9M2I6_ACYPI Uncharacterized protein OS=Acyrthosiphon pisum OX=7029 PE=3 SV=2
Query_10010	tr|X1WJM1|X1WJM1_ACYPI Uncharacterized protein OS=Acyrthosiphon pisum OX=7029 PE=3 SV=1
Query_10011	tr|Q17MA9|Q17MA9_AEDAE AAEL001061-PA OS=Aedes aegypti OX=7159 GN=GSTD1 PE=4 SV=1
Query_10012	tr|Q174V0|Q174V0_AEDAE AAEL006764-PA (Fragment) OS=Aedes aegypti OX=7159 GN=AAEL006764 PE=4 SV=1
Query_10013	tr|Q17MB0|Q17MB0_AEDAE AAEL001078-PA OS=Aedes aegypti OX=7159 GN=GSTD2 PE=4 SV=1
Query_10014	tr|Q17MB7|Q17MB7_AEDAE AAEL001059-PA OS=Aedes aegypti OX=7159 GN=GSTD3 PE=3 SV=1
Query_10015	tr|Q17MB8|Q17MB8_AEDAE AAEL001054-PA (Fragment) OS=Aedes aegypti OX=7159 GN=GSTD4 PE=4 SV=2
Query_10016	tr|Q17MB1|Q17MB1_AEDAE AAEL001071-PA OS=Aedes aegypti OX=7159 GN=GSTD5 PE=4 SV=1
Query_10017	tr|Q16SH6|Q16SH6_AEDAE AAEL010591-PA OS=Aedes aegypti OX=7159 GN=GSTD6 PE=3 SV=1
Query_10018	tr|Q17MA8|Q17MA8_AEDAE AAEL001090-PA OS=Aedes aegypti OX=7159 GN=GSTD7 PE=4 SV=1
Query_10019	tr|Q170D2|Q170D2_AEDAE AAEL007954-PA OS=Aedes aegypti OX=7159 GN=GSTE1 PE=3 SV=2
Query_10020	tr|Q5PY77|Q5PY77_AEDAE AAEL007951-PA OS=Aedes aegypti OX=7159 GN=GSTe2 PE=1 SV=1
Query_10021	tr|Q170C6|Q170C6_AEDAE AAEL007947-PA OS=Aedes aegypti OX=7159 GN=GSTE3 PE=3 SV=1
Query_10022	tr|Q5PY78|Q5PY78_AEDAE AAEL007962-PA OS=Aedes aegypti OX=7159 GN=GSTe4 PE=2 SV=1
Query_10023	tr|Q170C9|Q170C9_AEDAE AAEL007964-PA OS=Aedes aegypti OX=7159 GN=GSTE5 PE=3 SV=1
Query_10024	tr|Q170C7|Q170C7_AEDAE AAEL007946-PA OS=Aedes aegypti OX=7159 GN=GSTE6 PE=4 SV=2
Query_10025	tr|Q170C8|Q170C8_AEDAE AAEL007948-PA OS=Aedes aegypti OX=7159 GN=GSTE7 PE=3 SV=1

Query_10026	tr|Q170D3|Q170D3_AEDAE AAEL007955-PA (Fragment) OS=Aedes aegypti OX=7159 GN=GSTE8 PE=4 SV=1
Query_10027	tr|Q5PY76|Q5PY76_AEDAE AAEL009017-PA (Fragment) OS=Aedes aegypti OX=7159 GN=GSTt1 PE=2 SV=1
Query_10028	tr|Q16X19|Q16X19_AEDAE AAEL009016-PA OS=Aedes aegypti OX=7159 GN=GSTT2 PE=3 SV=2
Query_10029	tr|Q16X21|Q16X21_AEDAE AAEL009020-PA OS=Aedes aegypti OX=7159 GN=GSTT3 PE=3 SV=2
Query_10030	tr|Q17DF6|Q17DF6_AEDAE AAEL004229-PA OS=Aedes aegypti OX=7159 GN=GSTT4 PE=3 SV=1
Query_10031	tr|A0A023ENG1|A0A023ENG1_AEDAL Putative cpij002683 glutathione s-transferase 1-1 OS=Aedes albopictus OX=7160 GN=RP20_CCG018982 PE=2 SV=1
Query_10032	tr|A0A023EKA0|A0A023EKA0_AEDAL Uncharacterized protein OS=Aedes albopictus OX=7160 GN=109402247 PE=2 SV=1
Query_10033	tr|A0A182GCK7|A0A182GCK7_AEDAL Uncharacterized protein OS=Aedes albopictus OX=7160 GN=RP20_CCG007573 PE=3 SV=1
Query_10034	tr|A0A182GEB4|A0A182GEB4_AEDAL Uncharacterized protein OS=Aedes albopictus OX=7160 GN=RP20_CCG008439 PE=3 SV=1
Query_10035	tr|A0A182GVH4|A0A182GVH4_AEDAL Uncharacterized protein OS=Aedes albopictus OX=7160 GN=RP20_CCG016463 PE=3 SV=1
Query_10036	tr|A0A182GCK8|A0A182GCK8_AEDAL Uncharacterized protein OS=Aedes albopictus OX=7160 GN=109424803 PE=3 SV=1
Query_10037	tr|A0A023EK27|A0A023EK27_AEDAL Putative glutathione s-transferase OS=Aedes albopictus OX=7160 PE=2 SV=1
Query_10038	tr|A0A023EMF5|A0A023EMF5_AEDAL Putative glutathione s-transferase e4 OS=Aedes albopictus OX=7160 PE=2 SV=1
Query_10039	tr|A0A023EK11|A0A023EK11_AEDAL Putative glutathione s-transferase e2 OS=Aedes albopictus OX=7160 PE=2 SV=1
Query_10040	tr|A0A023EK53|A0A023EK53_AEDAL Putative glutathione s-transferase e4 OS=Aedes albopictus OX=7160 PE=2 SV=1
Query_10041	tr|A0A1W7R8Y6|A0A1W7R8Y6_AEDAL Putative glutathione s-transferase OS=Aedes albopictus OX=7160 PE=3 SV=1
Query_10042	tr|A0A023EJP7|A0A023EJP7_AEDAL Putative glutathione s-transferase 1 isoform d OS=Aedes albopictus OX=7160 PE=2 SV=1
Query_10043	tr|A0A182GJY7|A0A182GJY7_AEDAL Uncharacterized protein OS=Aedes albopictus OX=7160 GN=RP20_CCG011233 PE=3 SV=1
Query_10044	tr|A0A182GCK6|A0A182GCK6_AEDAL Uncharacterized protein OS=Aedes albopictus OX=7160 GN=RP20_CCG007572 PE=3 SV=1
Query_10045	tr|A0A182GEB7|A0A182GEB7_AEDAL Uncharacterized protein OS=Aedes albopictus OX=7160 GN=RP20_CCG008442 PE=3 SV=1
Query_10046	tr|A0A182H003|A0A182H003_AEDAL Uncharacterized protein OS=Aedes albopictus OX=7160 GN=RP20_CCG018985 PE=3 SV=1
Query_10047	tr|A0A182GYD0|A0A182GYD0_AEDAL Uncharacterized protein OS=Aedes albopictus OX=7160 GN=RP20_CCG018089 PE=3 SV=1
Query_10048	tr|A0A182GHZ1|A0A182GHZ1_AEDAL Uncharacterized protein OS=Aedes albopictus OX=7160 GN=RP20_CCG010237 PE=3 SV=1
Query_10049	tr|A0A182GYD1|A0A182GYD1_AEDAL Uncharacterized protein OS=Aedes albopictus OX=7160 GN=RP20_CCG018090 PE=3 SV=1
Query_10050	tr|A0A182GYC8|A0A182GYC8_AEDAL Uncharacterized protein OS=Aedes albopictus OX=7160 GN=RP20_CCG018087 PE=3 SV=1
Query_10051	tr|A0A182GYC5|A0A182GYC5_AEDAL Uncharacterized protein OS=Aedes albopictus OX=7160 GN=RP20_CCG018084 PE=3 SV=1
Query_10052	tr|A0A182H001|A0A182H001_AEDAL Uncharacterized protein OS=Aedes albopictus OX=7160 GN=RP20_CCG018983 PE=3 SV=1

Query_10053	tr|A0A182GYC6|A0A182GYC6_AEDAL Uncharacterized protein OS=Aedes albopictus OX=7160 GN=RP20_CCG018085 PE=3 SV=1
Query_10054	tr|A0A182G345|A0A182G345_AEDAL Uncharacterized protein OS=Aedes albopictus OX=7160 GN=RP20_CCG001852 PE=3 SV=1
Query_10055	tr|A0A182H002|A0A182H002_AEDAL Uncharacterized protein OS=Aedes albopictus OX=7160 GN=RP20_CCG018984 PE=3 SV=1
Query_10056	tr|A0A1W7R8X7|A0A1W7R8X7_AEDAL Putative glutathione s-transferase e4 (Fragment) OS=Aedes albopictus OX=7160 PE=4 SV=1
Query_10057	tr|A0A182GEB5|A0A182GEB5_AEDAL Uncharacterized protein OS=Aedes albopictus OX=7160 GN=RP20_CCG008440 PE=4 SV=1
Query_10058	tr|A0A182GYC7|A0A182GYC7_AEDAL Uncharacterized protein OS=Aedes albopictus OX=7160 GN=RP20_CCG018086 PE=4 SV=1
Query_10059	tr|A0A182GYC9|A0A182GYC9_AEDAL Uncharacterized protein OS=Aedes albopictus OX=7160 GN=RP20_CCG018088 PE=4 SV=1
Query_10060	tr|A0A182GEB6|A0A182GEB6_AEDAL Uncharacterized protein OS=Aedes albopictus OX=7160 PE=4 SV=1
Query_10061	XP_013192421.1 PREDICTED: glutathione S-transferase 1-like [Amyelois transitella]
Query_10062	XP_013189941.1 PREDICTED: glutathione S-transferase 1-like [Amyelois transitella]
Query_10063	XP_013196516.1 PREDICTED: glutathione S-transferase 1-like [Amyelois transitella]
Query_10064	XP_013187010.1 PREDICTED: glutathione S-transferase 1-like [Amyelois transitella]
Query_10065	XP_013196509.1 PREDICTED: glutathione S-transferase 1, isoform C-like [Amyelois transitella]
Query_10066	XP_013194108.1 PREDICTED: uncharacterized protein LOC106137747 [Amyelois transitella]
Query_10067	XP_013196508.1 PREDICTED: glutathione S-transferase 1-1-like [Amyelois transitella]
Query_10068	XP_013198047.1 PREDICTED: glutathione S-transferase 1-like [Amyelois transitella]
Query_10069	XP_013187696.1 PREDICTED: glutathione S-transferase 1, isoform C-like [Amyelois transitella]
Query_10070	XP_013182960.1 PREDICTED: glutathione S-transferase 1-like [Amyelois transitella]
Query_10071	NP_001299595.1 uncharacterized protein LOC106136399 precursor [Amyelois transitella]
Query_10072	XP_013187697.1 PREDICTED: glutathione S-transferase 1, isoform C-like [Amyelois transitella]
Query_10073	XP_013192447.1 PREDICTED: glutathione S-transferase 1-like [Amyelois transitella]
Query_10074	XP_013194098.1 PREDICTED: glutathione S-transferase 1-like [Amyelois transitella]
Query_10075	tr|W5JW71|W5JW71_ANODA Glutathione transferase, delta class OS=Anopheles darlingi OX=43151 GN=AND_000801 PE=3 SV=1
Query_10076	tr|W5JXC1|W5JXC1_ANODA Glutathione transferase, theta class OS=Anopheles darlingi OX=43151 GN=AND_000084 PE=3 SV=1
Query_10077	tr|W5JWL4|W5JWL4_ANODA Glutathione transferase, theta class OS=Anopheles darlingi OX=43151 GN=AND_000085 PE=3 SV=1
Query_10078	tr|W5JWI4|W5JWI4_ANODA Glutathione S-transferase 1-6 OS=Anopheles darlingi OX=43151 GN=AND_000834 PE=3 SV=1
Query_10079	tr|W5J8C0|W5J8C0_ANODA Glutathione transferase OS=Anopheles darlingi OX=43151 GN=AND_008209 PE=3 SV=1

Query_10080	tr|W5J9X2|W5J9X2_ANODA Glutathione S-transferase, epsilon class OS=Anopheles darlingi OX=43151 GN=AND_008205 PE=3 SV=1
Query_10081	tr|W5JTD5|W5JTD5_ANODA Glutathione transferase OS=Anopheles darlingi OX=43151 GN=AND_000803 PE=3 SV=1
Query_10082	tr|W5J8C4|W5J8C4_ANODA Glutathione S-transferase, epsilon class OS=Anopheles darlingi OX=43151 GN=AND_008208 PE=3 SV=1
Query_10083	tr|W5JA16|W5JA16_ANODA Glutathione S-transferase theta-2 OS=Anopheles darlingi OX=43151 GN=AND_008797 PE=3 SV=1
Query_10084	tr|W5J546|W5J546_ANODA Glutathione transferase OS=Anopheles darlingi OX=43151 GN=AND_008798 PE=3 SV=1
Query_10085	tr|W5JJP4|W5JJP4_ANODA Glutathione transferase, delta class OS=Anopheles darlingi OX=43151 GN=AND_004770 PE=3 SV=1
Query_10086	tr|W5JBQ5|W5JBQ5_ANODA Glutathione S-transferase, epsilon class OS=Anopheles darlingi OX=43151 GN=AND_008212 PE=3 SV=1
Query_10087	tr|W5JVI0|W5JVI0_ANODA Glutathione S transferase D1 OS=Anopheles darlingi OX=43151 GN=AND_000833 PE=3 SV=1
Query_10088	tr|W5J6X4|W5J6X4_ANODA Glutathione S-transferase, epsilon class OS=Anopheles darlingi OX=43151 GN=AND_008200 PE=3 SV=1
Query_10089	tr|W5JSQ1|W5JSQ1_ANODA Glutathione transferase OS=Anopheles darlingi OX=43151 GN=AND_000804 PE=4 SV=1
Query_10090	tr|A0A2M4CU45|A0A2M4CU45_ANODA Putative chain a glutathione s-transferase OS=Anopheles darlingi OX=43151 PE=3 SV=1
Query_10091	tr|A0A2M4CV43|A0A2M4CV43_ANODA Putative chain a glutathione s-transferase OS=Anopheles darlingi OX=43151 PE=3 SV=1
Query_10092	tr|A0A2M4CJX8|A0A2M4CJX8_ANODA Putative glutathione s-transferase 1 isoform d (Fragment) OS=Anopheles darlingi OX=43151 PE=3 SV=1
Query_10093	tr|A0A2M4CYI6|A0A2M4CYI6_ANODA Putative glutathione s-transferase epsilon class OS=Anopheles darlingi OX=43151 PE=3 SV=1
Query_10094	tr|W5JWK8|W5JWK8_ANODA Glutathione transferase OS=Anopheles darlingi OX=43151 GN=AND_000802 PE=4 SV=1
Query_10095	tr|W5J882|W5J882_ANODA Glutathione transferase OS=Anopheles darlingi OX=43151 GN=AND_008796 PE=4 SV=1
Query_10096	tr|W5JBI5|W5JBI5_ANODA Glutathione transferase OS=Anopheles darlingi OX=43151 GN=AND_008211 PE=4 SV=1
Query_10097	tr|W5J6X9|W5J6X9_ANODA Glutathione S-transferase, epsilon class OS=Anopheles darlingi OX=43151 GN=AND_008204 PE=4 SV=1
Query_10098	tr|W5JW53|W5JW53_ANODA Glutathione transferase, delta class OS=Anopheles darlingi OX=43151 GN=AND_000835 PE=4 SV=1
Query_10099	tr|W5JC52|W5JC52_ANODA Glutathione transferase, delta class OS=Anopheles darlingi OX=43151 GN=AND_007981 PE=4 SV=1
Query_10100	tr|A0A084WTH5|A0A084WTH5_ANOSI Glutathione S-transferase D6 OS=Anopheles sinensis OX=74873 GN=ZHAS_00021747 PE=3 SV=1
Query_10101	tr|A0A084VMZ2|A0A084VMZ2_ANOSI Glutathione s-transferase E5 OS=Anopheles sinensis OX=74873 GN=ZHAS_00006696 PE=3 SV=1
Query_10102	tr|A0A084WQQ1|A0A084WQQ1_ANOSI Glutathione transferase epsilon3 OS=Anopheles sinensis OX=74873 GN=ZHAS_00020795 PE=3 SV=1
Query_10103	tr|A0A084WQQ3|A0A084WQQ3_ANOSI Glutathione s-transferase E2 OS=Anopheles sinensis OX=74873 GN=ZHAS_00020699 PE=3 SV=1
Query_10104	tr|A0A084VQI2|A0A084VQI2_ANOSI AGAP000888-PA-like protein OS=Anopheles sinensis OX=74873 GN=ZHAS_00007603 PE=3 SV=1
Query_10105	tr|A0A084VQI3|A0A084VQI3_ANOSI Uncharacterized protein OS=Anopheles sinensis OX=74873 GN=ZHAS_00007604 PE=3 SV=1
Query_10106	tr|A0A084WTH9|A0A084WTH9_ANOSI AGAP004383-PA-like protein OS=Anopheles sinensis OX=74873 GN=ZHAS_00021752 PE=3 SV=1

Query_10107	tr|A0A084WTI1|A0A084WTI1_ANOSI Uncharacterized protein OS=Anopheles sinensis OX=74873 GN=ZHAS_00021754 PE=3 SV=1
Query_10108	tr|A0A084WTH8|A0A084WTH8_ANOSI AGAP004382-PA-like protein OS=Anopheles sinensis OX=74873 GN=ZHAS_00021751 PE=3 SV=1
Query_10109	tr|A0A084WTH6|A0A084WTH6_ANOSI AGAP004380-PA-like protein OS=Anopheles sinensis OX=74873 GN=ZHAS_00021748 PE=3 SV=1
Query_10110	tr|A0A084WTH7|A0A084WTH7_ANOSI AGAP004382-PA-like protein OS=Anopheles sinensis OX=74873 GN=ZHAS_00021749 PE=3 SV=1
Query_10111	tr|A0A084WTI2|A0A084WTI2_ANOSI AGAP004171-PA-like protein OS=Anopheles sinensis OX=74873 GN=ZHAS_00021755 PE=3 SV=1
Query_10112	tr|A0A084WTH4|A0A084WTH4_ANOSI AGAP004378-PA-like protein OS=Anopheles sinensis OX=74873 GN=ZHAS_00021746 PE=3 SV=1
Query_10113	tr|A0A084VUG1|A0A084VUG1_ANOSI AGAP003257-PA-like protein OS=Anopheles sinensis OX=74873 GN=ZHAS_00009226 PE=3 SV=1
Query_10114	tr|A0A084VMZ0|A0A084VMZ0_ANOSI AGAP009190-PA-like protein OS=Anopheles sinensis OX=74873 GN=ZHAS_00006694 PE=3 SV=1
Query_10115	tr|A0A084WTJ6|A0A084WTJ6_ANOSI AGAP004164-PB-like protein OS=Anopheles sinensis OX=74873 GN=ZHAS_00021770 PE=3 SV=1
Query_10116	tr|A0A084VMZ4|A0A084VMZ4_ANOSI Glutathione s-transferase E2 OS=Anopheles sinensis OX=74873 GN=ZHAS_00006698 PE=4 SV=1
Query_10117	tr|A0A084VMZ3|A0A084VMZ3_ANOSI Glutathione s-transferase E4 OS=Anopheles sinensis OX=74873 GN=ZHAS_00006697 PE=4 SV=1
Query_10118	tr|A0A084WQQ2|A0A084WQQ2_ANOSI Glutathione s-transferase E2 OS=Anopheles sinensis OX=74873 GN=ZHAS_00020698 PE=4 SV=1
Query_10119	tr|A0A084VMZ1|A0A084VMZ1_ANOSI Glutathione s-transferase E6 OS=Anopheles sinensis OX=74873 GN=ZHAS_00006695 PE=4 SV=1
Query_10120	tr|A0A084WCJ1|A0A084WCJ1_ANOSI AGAP011334-PA-like protein OS=Anopheles sinensis OX=74873 GN=ZHAS_00015997 PE=4 SV=1
Query_10121	tr|A0A084WTI0|A0A084WTI0_ANOSI AGAP004173-PA-like protein OS=Anopheles sinensis OX=74873 GN=ZHAS_00021753 PE=4 SV=1
Query_10122	tr|A0A084WTJ9|A0A084WTJ9_ANOSI AGAP004163-PB-like protein OS=Anopheles sinensis OX=74873 GN=ZHAS_00021774 PE=4 SV=1
Query_10123	sp|Q93113|GST1D_ANOGA Glutathione S-transferase 1, isoform D OS=Anopheles gambiae OX=7165 GN=GstD1 PE=1 SV=1
Query_10124	tr|Q7QA79|Q7QA79_ANOGA AGAP004383-PA OS=Anopheles gambiae OX=7165 GN=GSTD10 PE=4 SV=1
Query_10125	tr|Q8MUS1|Q8MUS1_ANOGA Glutathione S-transferase D11 OS=Anopheles gambiae OX=7165 GN=GSTd11 PE=2 SV=1
Query_10126	tr|Q9GPL6|Q9GPL6_ANOGA AGAP004380-PA OS=Anopheles gambiae OX=7165 GN=GSTD12 PE=2 SV=1
Query_10127	sp|Q94999|GSTT2_ANOGA Glutathione S-transferase 2 OS=Anopheles gambiae OX=7165 GN=GstD2 PE=3 SV=2
Query_10128	tr|Q7PQ95|Q7PQ95_ANOGA AGAP004382-PA OS=Anopheles gambiae OX=7165 GN=GSTD3 PE=3 SV=3
Query_10129	tr|Q5TT03|Q5TT03_ANOGA AGAP004381-PA OS=Anopheles gambiae OX=7165 GN=GSTD4 PE=3 SV=1
Query_10130	tr|Q7QB59|Q7QB59_ANOGA AGAP004173-PA OS=Anopheles gambiae OX=7165 GN=GSTD5 PE=4 SV=1
Query_10131	tr|Q8MUS2|Q8MUS2_ANOGA Glutathione S-transferase D6 (Fragment) OS=Anopheles gambiae OX=7165 GN=GSTd6 PE=2 SV=1
Query_10132	sp|O76483|GSTT7_ANOGA Glutathione S-transferase D7 OS=Anopheles gambiae OX=7165 GN=GstD7 PE=2 SV=1
Query_10133	tr|Q5TTE5|Q5TTE5_ANOGA AGAP004171-PA OS=Anopheles gambiae OX=7165 GN=GSTD8 PE=3 SV=2

Query_10134	tr|Q86D84|Q86D84_ANOGA AGAP004172-PA OS=Anopheles gambiae OX=7165 GN=GSTd9 PE=4 SV=1
Query_10135	tr|Q9GPL9|Q9GPL9_ANOGA AGAP009195-PA OS=Anopheles gambiae OX=7165 GN=GSTE1 PE=2 SV=1
Query_10136	tr|Q7PVS6|Q7PVS6_ANOGA AGAP009194-PA OS=Anopheles gambiae OX=7165 GN=GSTE2 PE=1 SV=3
Query_10137	tr|Q8WQJ9|Q8WQJ9_ANOGA AGAP009197-PA OS=Anopheles gambiae OX=7165 GN=GSTe3 PE=3 SV=1
Query_10138	tr|Q8WQJ8|Q8WQJ8_ANOGA AGAP009193-PA OS=Anopheles gambiae OX=7165 GN=GSTe4 PE=2 SV=1
Query_10139	tr|Q8WQJ7|Q8WQJ7_ANOGA AGAP009192-PA OS=Anopheles gambiae OX=7165 GN=GSTe5 PE=2 SV=1
Query_10140	tr|A0NG89|A0NG89_ANOGA AGAP009191-PA OS=Anopheles gambiae OX=7165 GN=GSTE6 PE=3 SV=1
Query_10141	tr|Q7PVS4|Q7PVS4_ANOGA AGAP009196-PA OS=Anopheles gambiae OX=7165 GN=GSTE7 PE=4 SV=3
Query_10142	tr|Q8WQJ5|Q8WQJ5_ANOGA AGAP009190-PA OS=Anopheles gambiae OX=7165 GN=GSTe8 PE=2 SV=1
Query_10143	tr|Q8MUQ1|Q8MUQ1_ANOGA AGAP000761-PA OS=Anopheles gambiae OX=7165 GN=gstT1 PE=2 SV=1
Query_10144	tr|Q8MUQ2|Q8MUQ2_ANOGA AGAP000888-PA OS=Anopheles gambiae OX=7165 GN=gstT2 PE=2 SV=1
Query_10145	tr|A0A087ZVS9|A0A087ZVS9_APIME Uncharacterized protein OS=Apis mellifera OX=7460 GN=GstT1 PE=3 SV=1
Query_10146	tr|A0A088AFL5|A0A088AFL5_APIME Uncharacterized protein OS=Apis mellifera OX=7460 GN=GstD1 PE=4 SV=1
Query_10147	tr|Q6IVB7|Q6IVB7_APILI Glutathione-S-transferase 1 (Fragment) OS=Apis mellifera ligustica OX=7469 GN=gst1 PE=2 SV=2
Query_10148	tr|Q2I0J5|Q2I0J5_BOMMO Glutathione S-transferase 3 OS=Bombyx mori OX=7091 GN=693114 PE=2 SV=1
Query_10149	tr|H9IYE6|H9IYE6_BOMMO Uncharacterized protein OS=Bombyx mori OX=7091 PE=3 SV=1
Query_10150	tr|O61996|O61996_BOMMO Glutathione S-transferase OS=Bombyx mori OX=7091 GN=692678 PE=1 SV=1
Query_10151	tr|H9JAU4|H9JAU4_BOMMO Uncharacterized protein OS=Bombyx mori OX=7091 PE=4 SV=1
Query_10152	tr|H9JK84|H9JK84_BOMMO Uncharacterized protein OS=Bombyx mori OX=7091 PE=3 SV=1
Query_10153	tr|H9JKA0|H9JKA0_BOMMO Uncharacterized protein OS=Bombyx mori OX=7091 PE=3 SV=1
Query_10154	tr|H9JKP2|H9JKP2_BOMMO Uncharacterized protein OS=Bombyx mori OX=7091 PE=3 SV=1
Query_10155	tr|H9JKP3|H9JKP3_BOMMO Uncharacterized protein OS=Bombyx mori OX=7091 PE=3 SV=1
Query_10156	BmoriGSTE7
Query_10157	tr|B0LB16|B0LB16_BOMMO Epsilon-class glutathione transferase OS=Bombyx mori OX=7091 GN=gste PE=2 SV=1
Query_10158	tr|A0A077D6E7|A0A077D6E7_CNAME Glutathione S-transferase delta 1 OS=Cnaphalocrocis medinalis OX=437488 PE=2 SV=1
Query_10159	tr|A0A077D817|A0A077D817_CNAME Glutathione S-transferase epsilon 1 OS=Cnaphalocrocis medinalis OX=437488 PE=2 SV=1
Query_10160	tr|A0A0A7KNK1|A0A0A7KNK1_CNAME Glutathione S-transferase delta 4 OS=Cnaphalocrocis medinalis OX=437488 PE=2 SV=1

Query_10161	tr|A0A077D9Y8|A0A077D9Y8_CNAME Glutathione S-transferase epsilon 2 OS=Cnaphalocrocis medinalis OX=437488 PE=2 SV=1
Query_10162	tr|A0A0A7KL70|A0A0A7KL70_CNAME Glutathione S-transferase epsilon 9 OS=Cnaphalocrocis medinalis OX=437488 PE=2 SV=1
Query_10163	tr|A0A0A7KQB0|A0A0A7KQB0_CNAME Glutathione S-transferase delta 3 OS=Cnaphalocrocis medinalis OX=437488 PE=2 SV=1
Query_10164	tr|A0A077D602|A0A077D602_CNAME Glutathione S-transferase OS=Cnaphalocrocis medinalis OX=437488 PE=2 SV=1
Query_10165	tr|A0A0A7KLA5|A0A0A7KLA5_CNAME Glutathione S-transferase epsilon 8 OS=Cnaphalocrocis medinalis OX=437488 PE=2 SV=1
Query_10166	tr|A0A077D820|A0A077D820_CNAME Glutathione S-transferase epsilon 6 (Fragment) OS=Cnaphalocrocis medinalis OX=437488 PE=2 SV=1
Query_10167	tr|A0A077D5Z1|A0A077D5Z1_CNAME Glutathione S-transferase epsilon 5 (Fragment) OS=Cnaphalocrocis medinalis OX=437488 PE=2 SV=1
Query_10168	tr|A0A077D5Y7|A0A077D5Y7_CNAME Glutathione S-transferase delta 2 OS=Cnaphalocrocis medinalis OX=437488 PE=2 SV=1
Query_10169	tr|A0A077DB43|A0A077DB43_CNAME Glutathione S-transferase epsilon 3 OS=Cnaphalocrocis medinalis OX=437488 PE=2 SV=1
Query_10170	tr|A0A077D6F2|A0A077D6F2_CNAME Glutathione S-transferase epsilon 4 OS=Cnaphalocrocis medinalis OX=437488 PE=2 SV=1
Query_10171	tr|A0A0A7KLF5|A0A0A7KLF5_CNAME Glutathione S-transferase epsilon 7 OS=Cnaphalocrocis medinalis OX=437488 PE=2 SV=1
Query_10172	tr|B0VZJ3|B0VZJ3_CULQU Glutathione S-transferase D2 OS=Culex quinquefasciatus OX=7176 GN=6031025 PE=3 SV=1
Query_10173	tr|B0W6A9|B0W6A9_CULQU Glutathione-s-transferase theta, gst OS=Culex quinquefasciatus OX=7176 GN=6033850 PE=4 SV=1
Query_10174	tr|B0W6B2|B0W6B2_CULQU Glutathione S-transferase 1-1 OS=Culex quinquefasciatus OX=7176 GN=6033854 PE=3 SV=1
Query_10175	tr|B0W6C3|B0W6C3_CULQU Glutathione S-transferase 1 OS=Culex quinquefasciatus OX=7176 GN=6033865 PE=4 SV=1
Query_10176	tr|B0W6C4|B0W6C4_CULQU Glutathione S-transferase 1 OS=Culex quinquefasciatus OX=7176 GN=6033866 PE=4 SV=1
Query_10177	tr|B0W6C5|B0W6C5_CULQU Glutathione S-transferase D7 OS=Culex quinquefasciatus OX=7176 GN=6033867 PE=3 SV=1
Query_10178	tr|B0W6C7|B0W6C7_CULQU Glutathione transferase I OS=Culex quinquefasciatus OX=7176 GN=6033869 PE=3 SV=1
Query_10179	tr|B0W6C8|B0W6C8_CULQU Glutathione S-transferase theta-2 OS=Culex quinquefasciatus OX=7176 GN=6033870 PE=3 SV=1
Query_10180	tr|B0W6C9|B0W6C9_CULQU Glutathione S-transferase OS=Culex quinquefasciatus OX=7176 GN=6033871 PE=4 SV=1
Query_10181	tr|B0W6D0|B0W6D0_CULQU Glutathione S-transferase OS=Culex quinquefasciatus OX=7176 GN=6033872 PE=3 SV=1
Query_10182	tr|A0A1S4J718|A0A1S4J718_CULQU GSTD2 protein OS=Culex quinquefasciatus OX=7176 PE=3 SV=1
Query_10183	tr|B0W6D2|B0W6D2_CULQU Glutathione S-transferase 1-1 OS=Culex quinquefasciatus OX=7176 GN=6033874 PE=3 SV=1
Query_10184	tr|B0WQW9|B0WQW9_CULQU Glutathione S-transferase 1 OS=Culex quinquefasciatus OX=7176 GN=6041912 PE=3 SV=1
Query_10185	tr|B0WUG8|B0WUG8_CULQU Glutathione S-transferase 1-5 OS=Culex quinquefasciatus OX=7176 GN=6043364 PE=3 SV=1
Query_10186	tr|B0X3C7|B0X3C7_CULQU Glutathione-s-transferase theta, gst OS=Culex quinquefasciatus OX=7176 GN=6047041 PE=3 SV=1
Query_10187	tr|B0X3C8|B0X3C8_CULQU Glutathione-s-transferase theta, gst OS=Culex quinquefasciatus OX=7176 GN=6047042 PE=3 SV=1

Query_10188	tr|B0X3C9|B0X3C9_CULQU Glutathione-s-transferase theta, gst OS=Culex quinquefasciatus OX=7176 GN=6047043 PE=3 SV=1
Query_10189	tr|B0X3D0|B0X3D0_CULQU Glutathione S-transferase theta-1 OS=Culex quinquefasciatus OX=7176 GN=6047044 PE=3 SV=1
Query_10190	tr|B0XGJ6|B0XGJ6_CULQU Glutathione-s-transferase theta, gst OS=Culex quinquefasciatus OX=7176 GN=6052508 PE=3 SV=1
Query_10191	tr|B0XGJ7|B0XGJ7_CULQU Glutathione-s-transferase theta, gst OS=Culex quinquefasciatus OX=7176 GN=6052509 PE=4 SV=1
Query_10192	tr|B0XGJ8|B0XGJ8_CULQU Glutathione S-transferase 1-1 OS=Culex quinquefasciatus OX=7176 GN=6052510 PE=3 SV=1
Query_10193	tr|B0XGJ9|B0XGJ9_CULQU Glutathione S-transferase E2 OS=Culex quinquefasciatus OX=7176 GN=6052511 PE=4 SV=1
Query_10194	tr|B0XGK0|B0XGK0_CULQU Glutathione-s-transferase theta, gst OS=Culex quinquefasciatus OX=7176 GN=6052512 PE=3 SV=1
Query_10195	tr|B0XGK1|B0XGK1_CULQU Glutathione S-transferase 1-1 OS=Culex quinquefasciatus OX=7176 GN=6052513 PE=4 SV=1
Query_10196	tr|B0XGK2|B0XGK2_CULQU Glutathione-s-transferase theta, gst OS=Culex quinquefasciatus OX=7176 GN=6052515 PE=4 SV=1
Query_10197	tr|B0XGK3|B0XGK3_CULQU Glutathione-s-transferase theta, gst OS=Culex quinquefasciatus OX=7176 GN=6052516 PE=4 SV=1
Query_10198	tr|B0XGK4|B0XGK4_CULQU Glutathione-s-transferase theta OS=Culex quinquefasciatus OX=7176 GN=6052517 PE=4 SV=1
Query_10199	tr|B0XJU4|B0XJU4_CULQU Glutathione transferase AtGST OS=Culex quinquefasciatus OX=7176 GN=6053908 PE=4 SV=1
Query_10200	tr|B0XLC5|B0XLC5_CULQU Glutathione transferase AtGST OS=Culex quinquefasciatus OX=7176 GN=6054553 PE=3 SV=1
Query_10201	DPOGS200212-PA
Query_10202	DPOGS202622-PA
Query_10203	DPOGS204831-PA
Query_10204	DPOGS207576-PA
Query_10205	DPOGS207703-PA
Query_10206	DPOGS208312-PA
Query_10207	DPOGS209578-PA
Query_10208	DPOGS210477-PA
Query_10209	DPOGS210488-PA
Query_10210	DPOGS210526-PA
Query_10211	sp|P20432|GSTD1_DROME Glutathione S-transferase D1 OS=Drosophila melanogaster GN=GstD1 PE=1 SV=1
Query_10212	tr|Q9VGA1|Q9VGA1_DROME Glutathione S transferase D10, isoform A OS=Drosophila melanogaster GN=GstD10 PE=1 SV=1
Query_10213	tr|B7Z0S9|B7Z0S9_DROME Glutathione S transferase D11, isoform B OS=Drosophila melanogaster GN=GstD11 PE=4 SV=1
Query_10214	tr|Q8SXQ9|Q8SXQ9_DROME Glutathione S transferase D11, isoform A OS=Drosophila melanogaster GN=GstD11 PE=2 SV=1
Query_10215	sp|Q9VG98|GSTD2_DROME Glutathione S-transferase D2 OS=Drosophila melanogaster GN=GstD2 PE=1 SV=1
Query_10216	sp|Q9VG97|GSTD3_DROME Inactive glutathione S-transferase D3 OS=Drosophila melanogaster GN=GstD3 PE=2 SV=1

Query_10217	sp|Q9VG96|GSTD4_DROME Glutathione S-transferase D4 OS=Drosophila melanogaster GN=GstD4 PE=1 SV=1
Query_10218	sp|Q9VG95|GSTD5_DROME Glutathione S-transferase D5 OS=Drosophila melanogaster GN=GstD5 PE=1 SV=2
Query_10219	sp|Q9VG94|GSTD6_DROME Glutathione S-transferase D6 OS=Drosophila melanogaster GN=GstD6 PE=1 SV=1
Query_10220	sp|Q9VG93|GSTD7_DROME Glutathione S-transferase D7 OS=Drosophila melanogaster GN=GstD7 PE=1 SV=1
Query_10221	tr|Q9VG92|Q9VG92_DROME Glutathione S transferase D8 OS=Drosophila melanogaster GN=GstD8 PE=2 SV=1
Query_10222	tr|Q9VGA0|Q9VGA0_DROME Glutathione S transferase D9, isoform A OS=Drosophila melanogaster GN=GstD9 PE=1 SV=1
Query_10223	tr|Q7KK90|Q7KK90_DROME GH14654p OS=Drosophila melanogaster GN=GstE1 PE=1 SV=1
Query_10224	tr|Q4V6J1|Q4V6J1_DROME Glutathione S transferase E10, isoform A OS=Drosophila melanogaster GN=GstE10 PE=2 SV=1
Query_10225	tr|Q7JVZ8|Q7JVZ8_DROME Glutathione S transferase E11, isoform A OS=Drosophila melanogaster GN=GstE11 PE=1 SV=1
Query_10226	tr|Q9XYZ9|Q9XYZ9_DROME Glutathione S transferase E12, isoform A OS=Drosophila melanogaster GN=GstE12 PE=1 SV=1
Query_10227	tr|Q7JVI6|Q7JVI6_DROME Glutathione S transferase E13, isoform A OS=Drosophila melanogaster GN=GstE13 PE=1 SV=1
Query_10228	sp|Q7JYX0|GSTEE_DROME Glutathione S-transferase E14 OS=Drosophila melanogaster GN=GstE14 PE=1 SV=1
Query_10229	tr|Q7JYZ9|Q7JYZ9_DROME Glutathione S transferase E2 OS=Drosophila melanogaster GN=GstE2 PE=2 SV=1
Query_10230	tr|A1ZB68|A1ZB68_DROME FI01423p OS=Drosophila melanogaster GN=GstE3 PE=1 SV=1
Query_10231	tr|A1ZB69|A1ZB69_DROME Glutathione S transferase E4 OS=Drosophila melanogaster GN=GstE4 PE=3 SV=1
Query_10232	tr|A1ZB70|A1ZB70_DROME Glutathione S transferase E5 OS=Drosophila melanogaster GN=GstE5 PE=3 SV=1
Query_10233	tr|A1ZB71|A1ZB71_DROME Glutathione S transferase E6 OS=Drosophila melanogaster GN=GstE6 PE=1 SV=1
Query_10234	tr|A1ZB72|A1ZB72_DROME Glutathione S transferase E7 OS=Drosophila melanogaster GN=GstE7 PE=1 SV=1
Query_10235	tr|A1ZB73|A1ZB73_DROME Glutathione S transferase E8, isoform A OS=Drosophila melanogaster GN=GstE8 PE=3 SV=1
Query_10236	tr|Q7K8X7|Q7K8X7_DROME Glutathione S transferase E9 OS=Drosophila melanogaster GN=GstE9 PE=1 SV=1
Query_10237	tr|Q7K0B6|Q7K0B6_DROME Glutathione S transferase T1 OS=Drosophila melanogaster OX=7227 GN=GstT1 PE=1 SV=1
Query_10238	tr|A1Z7X7|A1Z7X7_DROME Glutathione S transferase T2 OS=Drosophila melanogaster OX=7227 GN=GstT2 PE=1 SV=2
Query_10239	tr|Q9VRA4|Q9VRA4_DROME Glutathione S transferase T3, isoform A OS=Drosophila melanogaster OX=7227 GN=GstT3 PE=3 SV=2
Query_10240	tr|E1JJS1|E1JJS1_DROME Glutathione S transferase T3, isoform B OS=Drosophila melanogaster OX=7227 GN=GstT3 PE=3 SV=1
Query_10241	tr|Q8MRM0|Q8MRM0_DROME GH16740p OS=Drosophila melanogaster OX=7227 GN=GstT4 PE=1 SV=1
Query_10242	tr|A0A291ARU4|A0A291ARU4_HELAM Glutathione S-transferase OS=Helicoverpa armigera OX=29058 GN=GST8 PE=2 SV=1
Query_10243	tr|A0A2W1BRB0|A0A2W1BRB0_HELAM Uncharacterized protein OS=Helicoverpa armigera OX=29058 GN=HaOG200226 PE=3 SV=1

Query_10244	tr|A0A2W1BSA5|A0A2W1BSA5_HELAM Uncharacterized protein OS=Helicoverpa armigera OX=29058 GN=HaOG200219 PE=3 SV=1
Query_10245	tr|A0A2W1BUA9|A0A2W1BUA9_HELAM Uncharacterized protein OS=Helicoverpa armigera OX=29058 GN=HaOG200217 PE=3 SV=1
Query_10246	tr|A0A2W1BZ02|A0A2W1BZ02_HELAM Uncharacterized protein OS=Helicoverpa armigera OX=29058 GN=HaOG200220 PE=3 SV=1
Query_10247	tr|A0MSN0|A0MSN0_HELAM Glutathione S-transferase OS=Helicoverpa armigera OX=29058 PE=2 SV=1
Query_10248	tr|B6A8L4|B6A8L4_HELAM Glutathione S-transferase (Fragment) OS=Helicoverpa armigera OX=29058 PE=2 SV=1
Query_10249	tr|C8YL89|C8YL89_HELAM Glutathione S-transferase 16 OS=Helicoverpa armigera OX=29058 GN=GST16 PE=2 SV=1
Query_10250	tr|D7NI45|D7NI45_HELAM Glutathione S-transferase OS=Helicoverpa armigera OX=29058 GN=GST6 PE=2 SV=1
Query_10251	AIB07715.1 glutathione S-transferase GSTD1 [Helicoverpa armigera armigera]
Query_10252	AIB07714.1 glutathione S-transferase GSTD3 [Helicoverpa armigera armigera]
Query_10253	AIB07716.1 glutathione S-transferase GSTD4 [Helicoverpa armigera armigera]
Query_10254	AIB07717.1 glutathione S-transferase GSTD5, partial [Helicoverpa armigera armigera]
Query_10255	XP_021189521.1 glutathione S-transferase D7-like isoform X1 [Helicoverpa armigera]
Query_10256	Helicoverpa_armigera_GSTE14-like_isoform_X2
Query_10257	tr|Q7Z0Q7|Q7Z0Q7_HELAM Glutathione S-transferase (Fragment) OS=Helicoverpa armigera OX=29058 PE=2 SV=1
Query_10258	tr|Q7Z0Q8|Q7Z0Q8_HELAM Glutathione S-transferase (Fragment) OS=Helicoverpa armigera OX=29058 PE=2 SV=1
Query_10259	tr|Q7Z0Q9|Q7Z0Q9_HELAM Glutathione S-transferase (Fragment) OS=Helicoverpa armigera OX=29058 PE=2 SV=1
Query_10260	tr|A0A2A4JDE9|A0A2A4JDE9_HELVI Uncharacterized protein OS=Heliothis virescens OX=7102 GN=B5V51_3669 PE=3 SV=1
Query_10261	tr|A0A2A4IXQ2|A0A2A4IXQ2_HELVI Uncharacterized protein OS=Heliothis virescens OX=7102 GN=B5V51_11472 PE=3 SV=1
Query_10262	tr|A0A2A4JI76|A0A2A4JI76_HELVI Uncharacterized protein OS=Heliothis virescens OX=7102 GN=B5V51_1534 PE=3 SV=1
Query_10263	tr|A0A2A4JDN1|A0A2A4JDN1_HELVI Uncharacterized protein OS=Heliothis virescens OX=7102 GN=B5V51_3670 PE=3 SV=1
Query_10264	tr|A0A2A4JU40|A0A2A4JU40_HELVI Uncharacterized protein OS=Heliothis virescens OX=7102 GN=B5V51_11931 PE=3 SV=1
Query_10265	tr|A0A2A4IV17|A0A2A4IV17_HELVI Uncharacterized protein OS=Heliothis virescens OX=7102 GN=B5V51_12180 PE=3 SV=1
Query_10266	tr|A0A2A4IXK8|A0A2A4IXK8_HELVI Uncharacterized protein OS=Heliothis virescens OX=7102 GN=B5V51_10494 PE=3 SV=1
Query_10267	tr|A0A2A4JCV1|A0A2A4JCV1_HELVI Uncharacterized protein OS=Heliothis virescens OX=7102 GN=B5V51_3945 PE=3 SV=1
Query_10268	tr|A0A2A4JV70|A0A2A4JV70_HELVI Uncharacterized protein OS=Heliothis virescens OX=7102 GN=B5V51_11943 PE=3 SV=1
Query_10269	tr|A0A2A4IY34|A0A2A4IY34_HELVI Uncharacterized protein (Fragment) OS=Heliothis virescens OX=7102 GN=B5V51_11290 PE=4 SV=1
Query_10270	tr|A0A2A4JKT9|A0A2A4JKT9_HELVI Uncharacterized protein OS=Heliothis virescens OX=7102 GN=B5V51_845 PE=4 SV=1
Query_10271	NP_001165913.1 glutathione S-transferase D1 [Nasonia vitripennis]

Query_10272	NP_001165914.1 glutathione S-transferase D3 [Nasonia vitripennis]
Query_10273	NP_001165915.1 glutathione S-transferase D5 [Nasonia vitripennis]
Query_10274	NP_001165925.1 glutathione S-transferase T2 [Nasonia vitripennis]
Query_10275	NP_001165926.1 glutathione S-transferase T1 [Nasonia vitripennis]
Query_10276	NP_001165927.1 glutathione S-transferase T3 [Nasonia vitripennis]
Query_10277	XP_001600187.1 PREDICTED: glutathione S-transferase D7 [Nasonia vitripennis]
Query_10278	tr|A0A0L7KM86|A0A0L7KM86_9NEOP Glutathione S-transferase OS=Operophtera brumata OX=104452 GN=OBRU01_24524 PE=3 SV=1
Query_10279	tr|A0A0L7LFQ3|A0A0L7LFQ3_9NEOP Glutathione S-transferase epsilon (Fragment) OS=Operophtera brumata OX=104452 GN=OBRU01_09353 PE=4 SV=1
Query_10280	tr|A0A0L7LME0|A0A0L7LME0_9NEOP Glutathione S-transferase 1-6 (Fragment) OS=Operophtera brumata OX=104452 GN=OBRU01_05580 PE=3 SV=1
Query_10281	tr|A0A0L7LPT4|A0A0L7LPT4_9NEOP Glutathione S-transferase OS=Operophtera brumata OX=104452 GN=OBRU01_04185 PE=3 SV=1
Query_10282	tr|A0A0L7LTB4|A0A0L7LTB4_9NEOP Glutathione S-transferase OS=Operophtera brumata OX=104452 GN=OBRU01_01600 PE=3 SV=1
Query_10283	tr|A0A0L7LTL7|A0A0L7LTL7_9NEOP Glutathione S-transferase epsilon 11 OS=Operophtera brumata OX=104452 GN=OBRU01_01599 PE=3 SV=1
Query_10284	tr|A0A0L7LI53|A0A0L7LI53_9NEOP Glutathione S-transferase epsilon 6 (Fragment) OS=Operophtera brumata OX=104452 GN=OBRU01_07826 PE=4 SV=1
Query_10285	tr|A0A0L7KLM3|A0A0L7KLM3_9NEOP Glutathione S-transferase 10 OS=Operophtera brumata OX=104452 GN=OBRU01_24503 PE=4 SV=1
Query_10286	tr|A0A0L7L1M8|A0A0L7L1M8_9NEOP Glutathione S-transferase epsilon 3 OS=Operophtera brumata OX=104452 GN=OBRU01_16992 PE=4 SV=1
Query_10287	tr|A0A0L7LM54|A0A0L7LM54_9NEOP Glutathione S-transferase 1 OS=Operophtera brumata OX=104452 GN=OBRU01_05579 PE=4 SV=1
Query_10288	tr|A0A194QXR6|A0A194QXR6_PAPMA Glutathione S-transferase D7 OS=Papilio machaon OX=76193 GN=RR48_13110 PE=4 SV=1
Query_10289	tr|A0A194R5D8|A0A194R5D8_PAPMA Glutathione S-transferase 1 OS=Papilio machaon OX=76193 GN=RR48_10522 PE=4 SV=1
Query_10290	tr|A0A0N1PJW2|A0A0N1PJW2_PAPMA Glutathione S-transferase theta-1 OS=Papilio machaon OX=76193 GN=RR48_04090 PE=3 SV=1
Query_10291	tr|A0A194QX62|A0A194QX62_PAPMA Glutathione S-transferase 1, isoform D OS=Papilio machaon OX=76193 GN=RR48_13184 PE=3 SV=1
Query_10292	tr|A0A194REA6|A0A194REA6_PAPMA Glutathione S-transferase 1-1 OS=Papilio machaon OX=76193 GN=RR48_07011 PE=3 SV=1
Query_10293	XP_014368559.1 PREDICTED: glutathione S-transferase E14-like [Papilio machaon]
Query_10294	tr|I4DRD9|I4DRD9_PAPPL Glutathione S transferase D8 OS=Papilio polytes OX=76194 PE=2 SV=1
Query_10295	tr|I4DRZ4|I4DRZ4_PAPPL Glutathione S transferase E8 (Fragment) OS=Papilio polytes OX=76194 PE=2 SV=1
Query_10296	XP_013145092.1 PREDICTED: glutathione S-transferase theta-1-like [Papilio polytes]
Query_10297	XP_013142369.1 PREDICTED: glutathione S-transferase 1, isoform D-like [Papilio polytes]
Query_10298	XP_013142272.1 PREDICTED: glutathione S-transferase D7-like isoform X2 [Papilio polytes]

Query_10299	Papilio_polytes_GST1-1-like_isoform_X1
Query_10300	tr|A0A194PX26|A0A194PX26_PAPXU Glutathione S-transferase theta-1 OS=Papilio xuthus OX=66420 GN=RR46_11408 PE=3 SV=1
Query_10301	tr|Q4R1I6|Q4R1I6_PAPXU Glutathione-S-transferase OS=Papilio xuthus OX=66420 GN=GST-pxcs1 PE=3 SV=1
Query_10302	tr|I4DKS5|I4DKS5_PAPXU Glutathionetransferase OS=Papilio xuthus OX=66420 PE=2 SV=1
Query_10303	tr|A0A194QHF9|A0A194QHF9_PAPXU Glutathione S-transferase 1-1 OS=Papilio xuthus OX=66420 GN=RR46_01739 PE=3 SV=1
Query_10304	tr|I4DNY4|I4DNY4_PAPXU Glutathionetransferase OS=Papilio xuthus OX=66420 PE=2 SV=1
Query_10305	tr|A0A194QG68|A0A194QG68_PAPXU Glutathione S-transferase D7 OS=Papilio xuthus OX=66420 GN=RR46_08238 PE=3 SV=1
Query_10306	tr|I4DQ12|I4DQ12_PAPXU Glutathione S transferase E6 (Fragment) OS=Papilio xuthus OX=66420 PE=2 SV=1
Query_10307	tr|E0VCV3|E0VCV3_PEDHC GSTD1-5 protein, putative OS=Pediculus humanus subsp. corporis OX=121224 GN=8238066 PE=4 SV=1
Query_10308	tr|E0VGM2|E0VGM2_PEDHC GSTD1-5 protein, putative OS=Pediculus humanus subsp. corporis OX=121224 GN=8240051 PE=3 SV=1
Query_10309	tr|E0VGM3|E0VGM3_PEDHC GSTD1-5 protein, putative OS=Pediculus humanus subsp. corporis OX=121224 GN=8240052 PE=3 SV=1
Query_10310	tr|E0VUR9|E0VUR9_PEDHC GSTD1-5 protein, putative OS=Pediculus humanus subsp. corporis OX=121224 GN=8230516 PE=3 SV=1
Query_10311	tr|A0A1P8L0T5|A0A1P8L0T5_PIERA Glutathione S-transferase theta 1 OS=Pieris rapae OX=64459 PE=2 SV=1
Query_10312	tr|A0A1P8L0U3|A0A1P8L0U3_PIERA Glutathione S-transferase epsilon 1 OS=Pieris rapae OX=64459 PE=2 SV=1
Query_10313	tr|A0A1P8L0T0|A0A1P8L0T0_PIERA Glutathione S-transferase epsilon 2 OS=Pieris rapae OX=64459 PE=2 SV=1
Query_10314	tr|A0A1P8L0T7|A0A1P8L0T7_PIERA Glutathione S-transferase OS=Pieris rapae OX=64459 PE=2 SV=1
Query_10315	tr|A0A1P8L0S7|A0A1P8L0S7_PIERA Glutathione S-transferase delta 2 OS=Pieris rapae OX=64459 PE=2 SV=1
Query_10316	tr|A0A1P8L0S9|A0A1P8L0S9_PIERA Glutathione S-transferase delta 1 OS=Pieris rapae OX=64459 PE=2 SV=1
Query_10317	tr|A0A1P8L0S2|A0A1P8L0S2_PIERA Glutathione S-transferase epsilon 3 OS=Pieris rapae OX=64459 PE=2 SV=1
Query_10318	XP_022126447.1 glutathione S-transferase E14-like [Pieris rapae]
Query_10319	tr|O77409|O77409_PLUXY Glutathione S-transferase isozyme 3 OS=Plutella xylostella OX=51655 GN=GST3 PE=2 SV=1
Query_10320	tr|Q2ABX5|Q2ABX5_PLUXY Glutathione S-Transferase-Epsilon7 OS=Plutella xylostella OX=51655 GN=GST3 PE=2 SV=1
Query_10321	tr|X5D044|X5D044_PLUXY Glutathione S-transferase (Fragment) OS=Plutella xylostella OX=51655 PE=2 SV=1
Query_10322	tr|X5CJS2|X5CJS2_PLUXY Glutathione S-transferase OS=Plutella xylostella OX=51655 PE=2 SV=1
Query_10323	tr|X5CYF6|X5CYF6_PLUXY Glutathione S-transferase OS=Plutella xylostella OX=51655 PE=2 SV=1
Query_10324	tr|X5CCB3|X5CCB3_PLUXY Glutathione S-transferase OS=Plutella xylostella OX=51655 PE=2 SV=1
Query_10325	tr|X5CYG4|X5CYG4_PLUXY Glutathione S-transferase OS=Plutella xylostella OX=51655 PE=2 SV=1
Query_10326	tr|X5CJR9|X5CJR9_PLUXY Glutathione S-transferase OS=Plutella xylostella OX=51655 PE=2 SV=1

Query_10327	tr|A0A1L8D6F5|A0A1L8D6F5_PLUXY Glutathione S-Transferase-Epsilon6 OS=Plutella xylostella OX=51655 PE=2 SV=1
Query_10328	tr|X5CHJ9|X5CHJ9_PLUXY Glutathione S-transferase OS=Plutella xylostella OX=51655 PE=2 SV=1
Query_10329	tr|D7URW9|D7URW9_PLUXY Glutathione S-transferase delta OS=Plutella xylostella OX=51655 GN=PxGSTd PE=2 SV=1
Query_10330	tr|X5CCB0|X5CCB0_PLUXY Glutathione S-transferase OS=Plutella xylostella OX=51655 PE=2 SV=1
Query_10331	tr|X5CJR6|X5CJR6_PLUXY Glutathione S-transferase OS=Plutella xylostella OX=51655 PE=2 SV=1
Query_10332	tr|X5D048|X5D048_PLUXY Glutathione S-transferase OS=Plutella xylostella OX=51655 PE=2 SV=1
Query_10333	tr|D2I931|D2I931_SPOLT Glutathione S-transferase epsilon 3 OS=Spodoptera litura OX=69820 PE=2 SV=1
Query_10334	tr|D2I930|D2I930_SPOLT Glutathione S-transferase epsilon 2 OS=Spodoptera litura OX=69820 PE=2 SV=1
Query_10335	tr|A0A075X2X0|A0A075X2X0_SPOLT Glutathione S-transferase theta 1 OS=Spodoptera litura OX=69820 PE=2 SV=1
Query_10336	tr|A0A075X3S8|A0A075X3S8_SPOLT Glutathione S-transferase delta 4 OS=Spodoptera litura OX=69820 PE=2 SV=1
Query_10337	tr|A0A075X2I2|A0A075X2I2_SPOLT Glutathione S-transferase epsilon 4 OS=Spodoptera litura OX=69820 PE=2 SV=1
Query_10338	tr|A0A075X2W2|A0A075X2W2_SPOLT Glutathione S-transferase epsilon 15 OS=Spodoptera litura OX=69820 PE=2 SV=1
Query_10339	tr|A0A075X8X2|A0A075X8X2_SPOLT Glutathione S-transferase epsilon 6 OS=Spodoptera litura OX=69820 PE=2 SV=1
Query_10340	tr|A0A075X8Y6|A0A075X8Y6_SPOLT Glutathione S-transferase delta 3 OS=Spodoptera litura OX=69820 PE=2 SV=1
Query_10341	tr|A0A075X244|A0A075X244_SPOLT Glutathione S-transferase epsilon 8 OS=Spodoptera litura OX=69820 PE=2 SV=1
Query_10342	tr|A0A075X8X7|A0A075X8X7_SPOLT Glutathione S-transferase epsilon 11 OS=Spodoptera litura OX=69820 PE=2 SV=1
Query_10343	tr|Q1EGY7|Q1EGY7_SPOLT Gst1 OS=Spodoptera litura OX=69820 GN=gst1 PE=2 SV=1
Query_10344	tr|A0A075X250|A0A075X250_SPOLT Glutathione S-transferase epsilon 13 (Fragment) OS=Spodoptera litura OX=69820 PE=2 SV=1
Query_10345	tr|A0A075X2W6|A0A075X2W6_SPOLT Glutathione S-transferase delta 2 (Fragment) OS=Spodoptera litura OX=69820 PE=2 SV=1
Query_10346	tr|A0A075X2V8|A0A075X2V8_SPOLT Glutathione S-transferase epsilon 10 (Fragment) OS=Spodoptera litura OX=69820 PE=2 SV=1
Query_10347	tr|A0A075X2J6|A0A075X2J6_SPOLT Glutathione S-transferase delta 1 (Fragment) OS=Spodoptera litura OX=69820 PE=2 SV=1
Query_10348	tr|A0A075X2I7|A0A075X2I7_SPOLT Glutathione S-transferase epsilon 9 OS=Spodoptera litura OX=69820 PE=2 SV=1
Query_10349	tr|A0A075X3R3|A0A075X3R3_SPOLT Glutathione S-transferase epsilon 7 (Fragment) OS=Spodoptera litura OX=69820 PE=2 SV=1
Query_10350	tr|A0A075X2V3|A0A075X2V3_SPOLT Glutathione S-transferase epsilon 5 OS=Spodoptera litura OX=69820 PE=2 SV=1
Query_10351	tr|A0A075X3R8|A0A075X3R8_SPOLT Glutathione S-transferase epsilon 12 OS=Spodoptera litura OX=69820 PE=2 SV=1
Query_10352	tr|A0A075X2J2|A0A075X2J2_SPOLT Glutathione S-transferase epsilon 14 OS=Spodoptera litura OX=69820 PE=2 SV=1
Query_10353	tr|A0A077D0A2|A0A077D0A2_SPOLT Glutathione S-transferase epsilon 3 (Fragment) OS=Spodoptera litura OX=69820 PE=2 SV=1

Query_10354	XP_022837684.1 glutathione S-transferase E14-like isoform X1 [Spodoptera litura]
Query_10355	TC003103_001 peptide: TC003103_001 pep:protein_coding
Query_10356	TC003104_001 peptide: TC003104_001 pep:protein_coding
Query_10357	TC003345_001 peptide: TC003345_001 pep:protein_coding
Query_10358	TC003347_001 peptide: TC003347_001 pep:protein_coding
Query_10359	TC003348_001 peptide: TC003348_001 pep:protein_coding
Query_10360	TC004442_001 peptide: TC004442_001 pep:protein_coding
Query_10361	TC004443_001 peptide: TC004443_001 pep:protein_coding
Query_10362	TC004444_001 peptide: TC004444_001 pep:protein_coding
Query_10363	TC004447_001 peptide: TC004447_001 pep:protein_coding
Query_10364	TC004448_001 peptide: TC004448_001 pep:protein_coding
Query_10365	TC004449_001 peptide: TC004449_001 pep:protein_coding
Query_10366	TC004450_001 peptide: TC004450_001 pep:protein_coding
Query_10367	TC004940_001 peptide: TC004940_001 pep:protein_coding
Query_10368	TC004941_001 peptide: TC004941_001 pep:protein_coding
Query_10369	TC004942_001 peptide: TC004942_001 pep:protein_coding
Query_10370	TC006215_001 peptide: TC006215_001 pep:protein_coding
Query_10371	TC009482_001 peptide: TC009482_001 pep:protein_coding
Query_10372	tr|A0A2W1BRE1|A0A2W1BRE1_HELAM Uncharacterized protein OS=Helicoverpa armigera OX=29058 GN=HaOG200227 PE=3 SV=1

Movie Legends

Movie 1. Trajectory of MD simulations of DmNobo[WT]_EST-GSH or DmNobo[Asp113Ala]_EST-GSH.

The Cα atoms of chain A of DmNobo[WT]_EST-GSH (blue) or DmNobo[Asp113Ala]_EST-GSH (white) in each frame were superimposed for 100 ns with those of DmNobo_EST-GSH at the initial state. The main chain of chain A of the protein is shown by a ribbon. Asp113 in DmNobo[WT]_EST-GSH, Ala113 in DmNobo[Asp113Ala]_EST-GSH, GSH, and EST are represented by sticks. Carbon atoms of EST in DmNobo[WT]_EST-GSH and those in DmNobo[Asp113Ala]_EST-GSH are colored in blue and magenta, respectively. The colors are the same as indicated in Fig. 4D.

Movie 2. An enlarged view of the trajectory of EST in MD simulations for DmNobo[WT]_EST-GSH or DmNobo[Asp113Ala]_EST-GSH.

An enlarged view of the trajectory of EST in MD simulations of DmNobo[WT]_EST-GSH or DmNobo[Asp113Ala]_EST-GSH (Movie 1)
